# Supplementary material for: Cell cycle gene regulation dynamics revealed by RNA velocity and deep-learning
Source: Nat Commun. 2022 May 23;13:2865. doi: 10.1038/s41467-022-30545-8 (PMC9126911; doi:10.1038/s41467-022-30545-8)
Supplement: Supplementary file 1 — Supplementary Information [file 41467_2022_30545_MOESM1_ESM.pdf]

Supplementary Information

Cell cycle gene regulation dynamics revealed by RNA velocity and deep-learning

Andrea Riba, Attila Oravecz, Matej Durik, Sara Jiménez, Violaine Alunni, Marie Cerciat, Matthieu Jung, Céline Keime, William M. Keyes, Nacho Molina

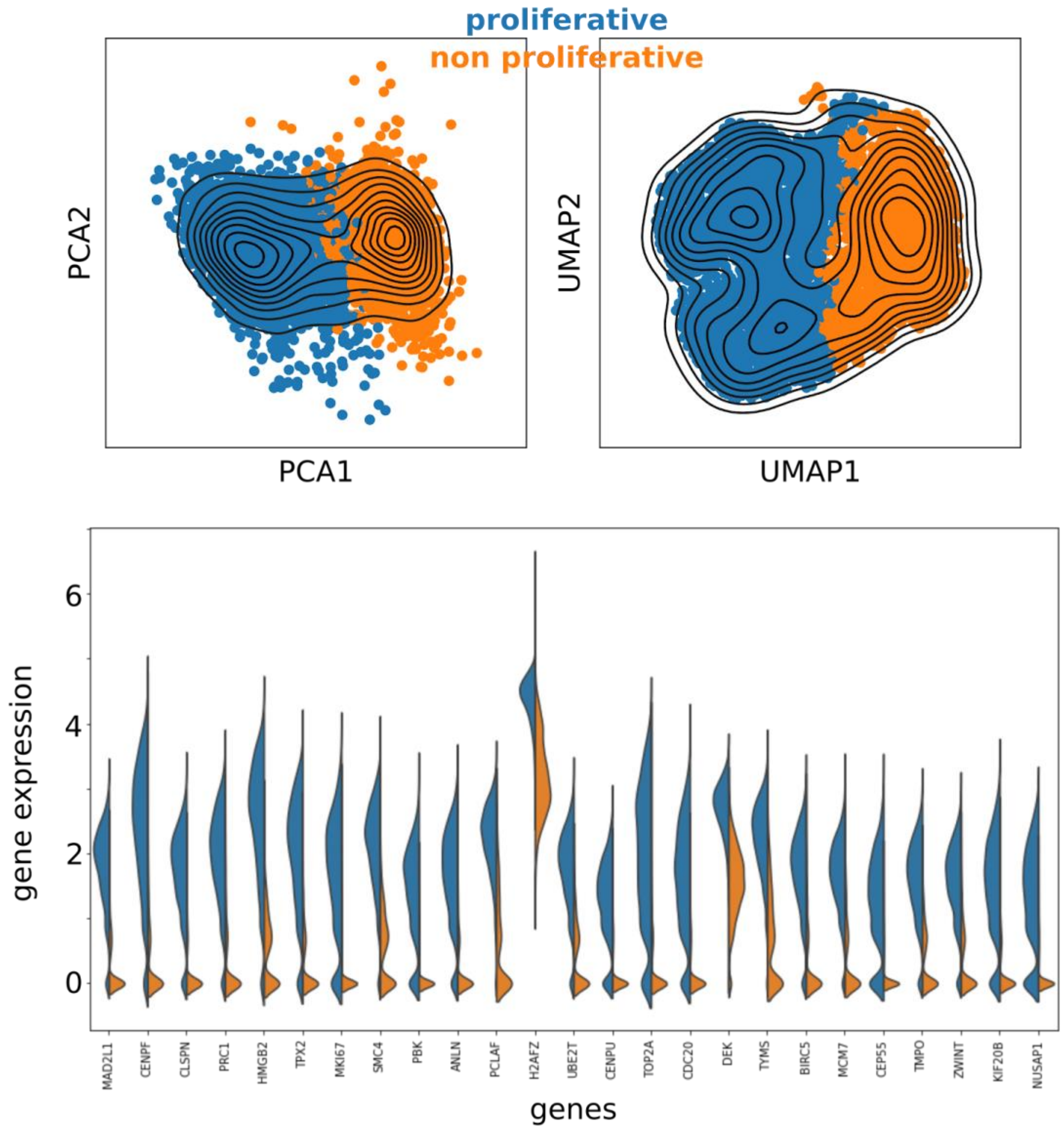

Supplementary Figure S1. Non proliferative subpopulation in the fibroblast dataset. The human fibroblasts dataset contains two subpopulations, one expressing cell cycle genes (blue) and the other not expressing them (orange). The two populations are distinguishable in the PCA and UMAP projections. Leiden clustering was performed to assign cells to the two subpopulations. The top genes identifying the proliferative cluster against the nonproliferative ones are mostly associated with mitosis (DAVID UP_Keywords Benjamini=1e-13) and cell cycle (DAVID UP_Keywords Benjamini=2e-15).

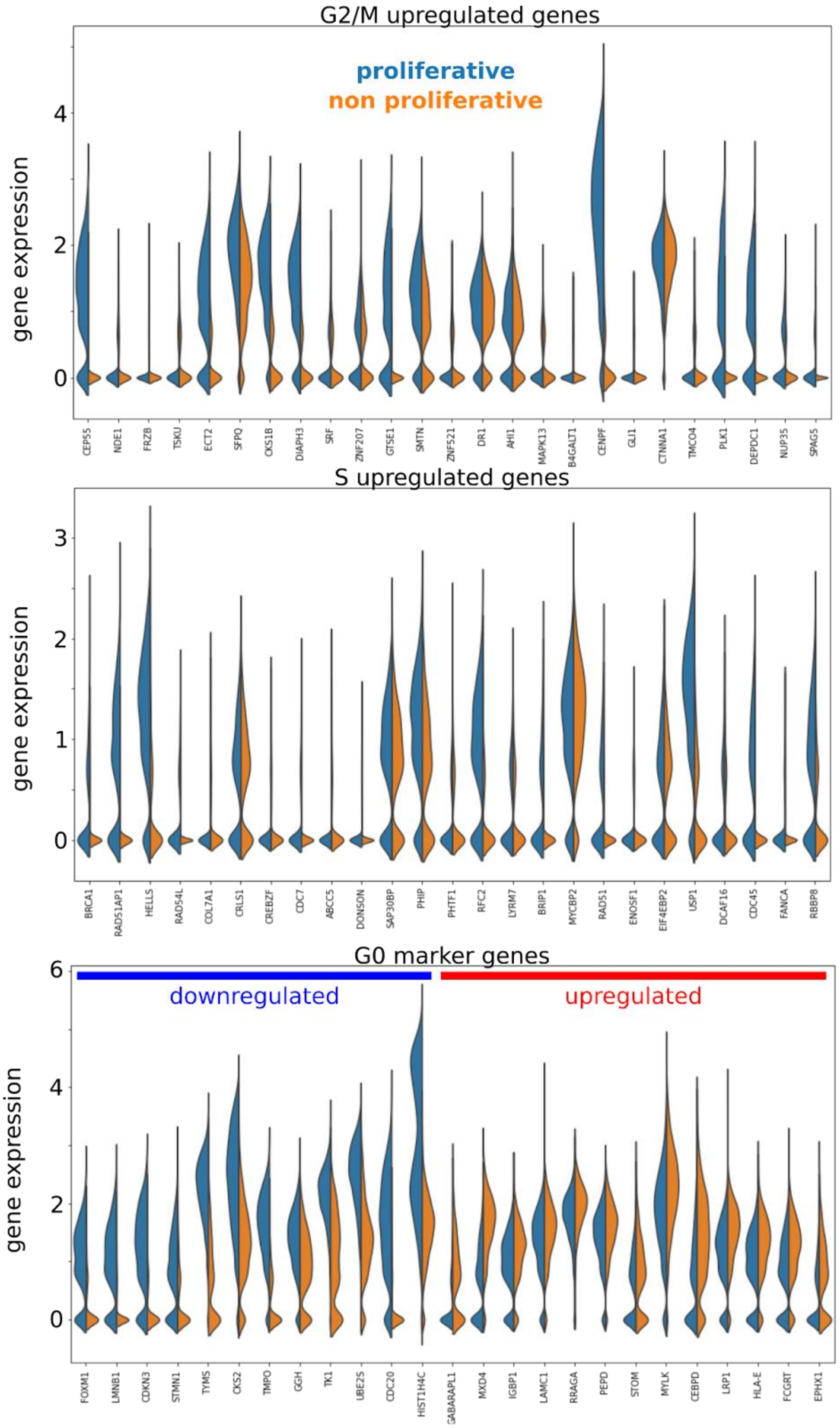

Supplementary Figure S2. The non proliferative subpopulation may be linked to a pre-quiescent state. The human fibroblasts dataset contains two subpopulations, as shown in Supplementary Figure S1. Examples of marker genes for G2/M, S and G0 phases are shown. G0 markers split in downregulated and upregulated genes and are taken from Coller et al. and Cheung and Rando [63,64]

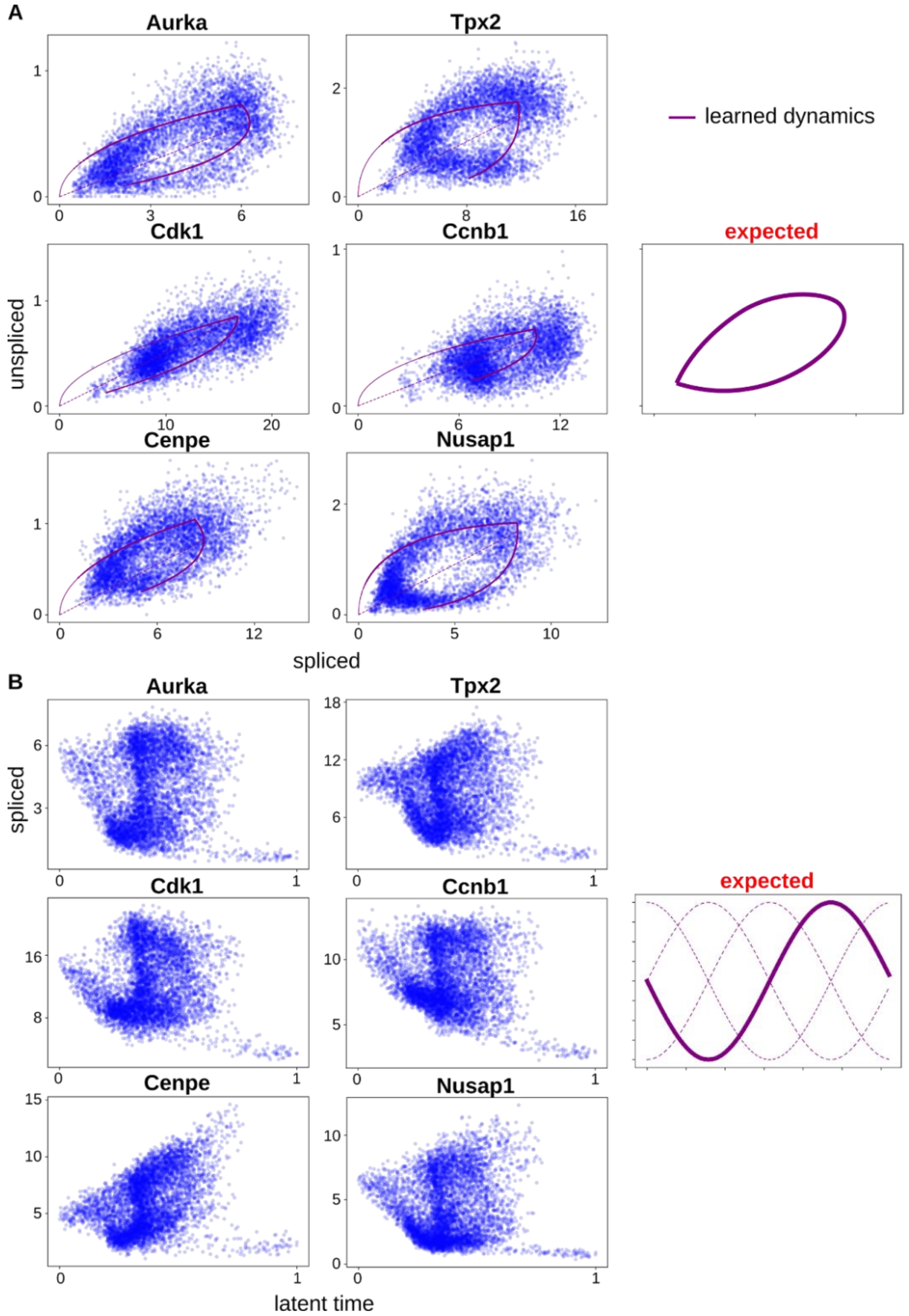

Supplementary Figure S3. Generalized RNA velocity (scVelo[19]) cannot fit the correct model and latent time. A. Examples of models learned by scVelo that do not fit the data correctly. The main issue seems to be related to the inability to capture the lower steady-state and, therefore, to close the activation-deactivation phases. **B.** The expression of the genes as a function of the latent time shows inconsistent patterns. None of the genes shows the

expected cyclical pattern that should be observed for cell-cycle genes, instead the gene expressions accumulate around latent time 0.5.

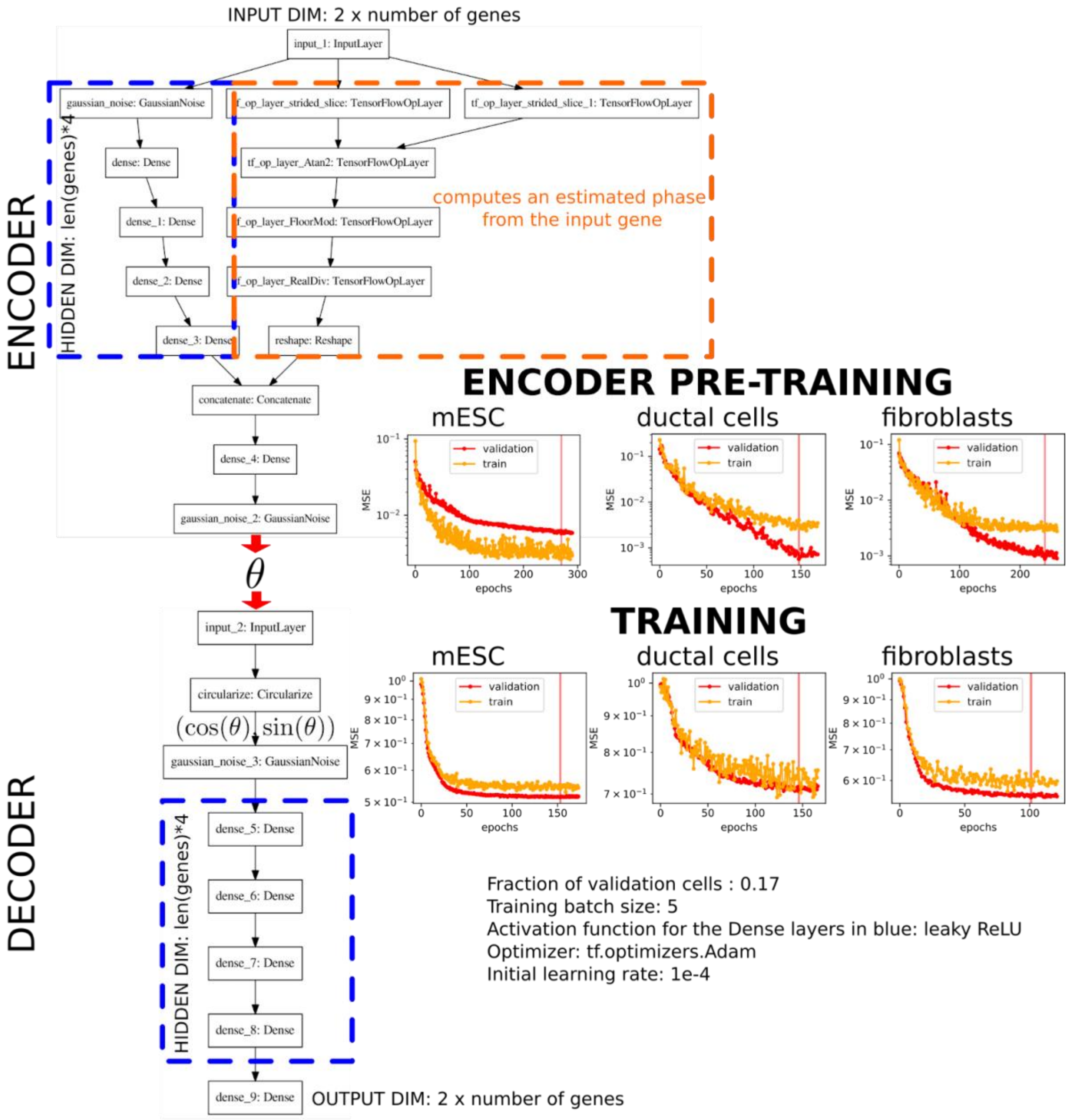

Supplementary Figure S4. DeepCycle - Autoencoder structure and training. The input and output layers of the autoencoder consist of densely connected layers of size twice the number of input genes. The densely connected layers in the blue boxes have a size of 4 times the number of genes and are activated through a leaky ReLU function. The orange box calculates the atan2 for the gene selected as input gene and concatenates this value with the output of the dense layers from the first part of the encoder. The concatenation is feeded to a Dense layer of size four times the number of genes and outputs a real number ($\boldsymbol{\theta}$). The real number is the input of the decoder that transforms it in ($\cos(\boldsymbol{\theta})$, $\sin(\boldsymbol{\theta})$) with the layer Circularize. The bidimensional vector is then fed to a series of densely connected layers till the output layer. The GaussianNoise layers add gaussian noise to the inputs to avoid the neural network overfitting the data.

The training is performed in 2 steps: 1) training the encoder on the phases estimated from the input gene (atan2 of z-scored spliced and unspliced reads); 2) training encoder+decoder to reconstruct the unspliced-spliced reads. Both training steps have an early stop when they reach a plateau *tf.keras.callbacks.EarlyStopping(monitor='val_loss', min_delta=0.0, patience=20, verbose=1, mode='auto', restore_best_weights=True)* and the learning rate decreases accordingly with *tf.keras.callbacks.ReduceLROnPlateau(monitor='val_loss', factor=0.8, patience=5, min_lr=0.00001)*. 17% of the input cells are used as validation set and the training is performed in batches of 5 cells. Stochastic Gradient Descent, RMSprop and Adam optimizers have been tested and Adam was the one giving the best performance. The optimization has been performed on the Mean Squared Error (MSE) between the input and the output.

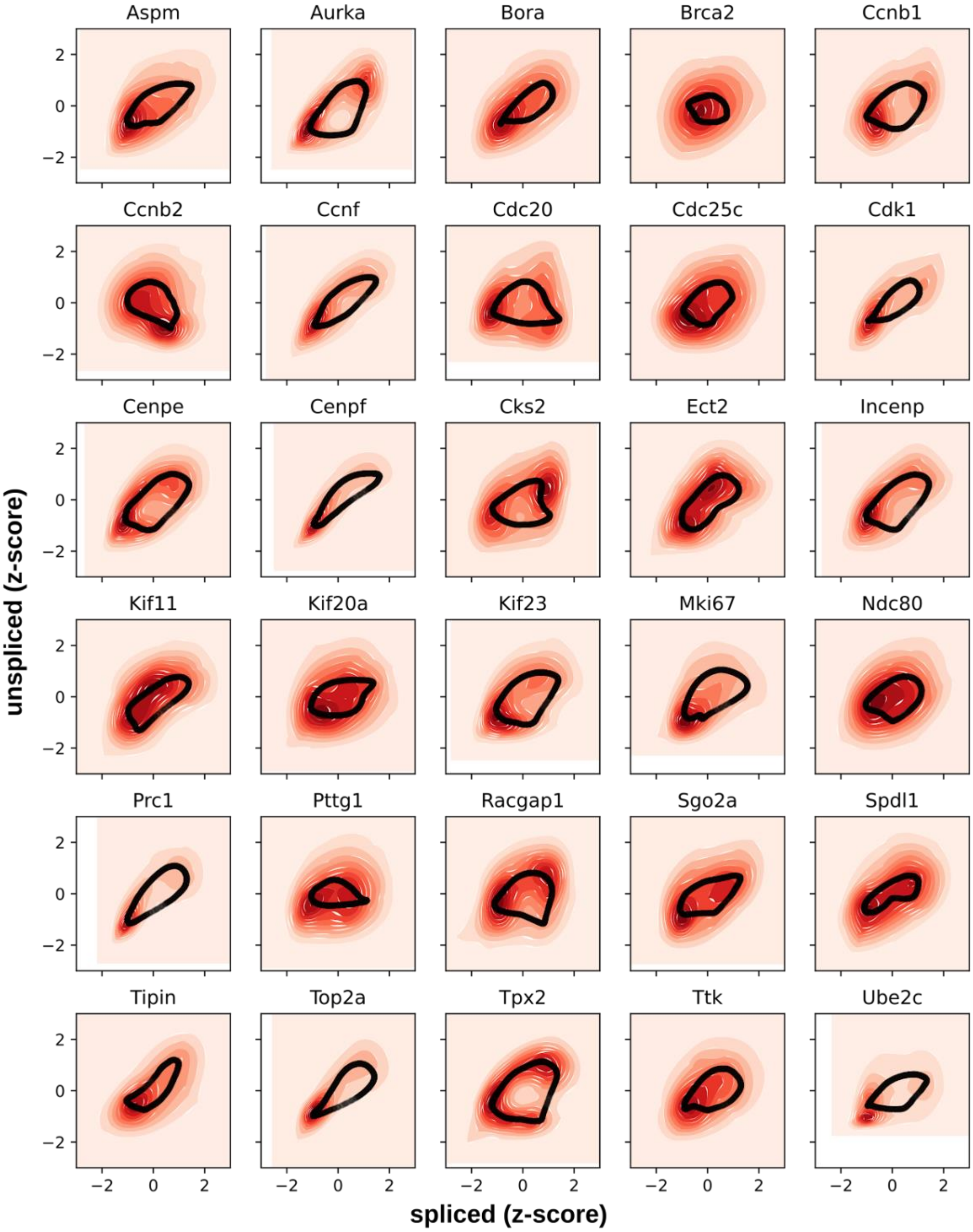

Supplementary Figure S5. Examples of fits for cycling genes from DeepCycle in the mESC dataset. Normalized expressions (z-scores) are fed into DeepCycle to extract the circular path.

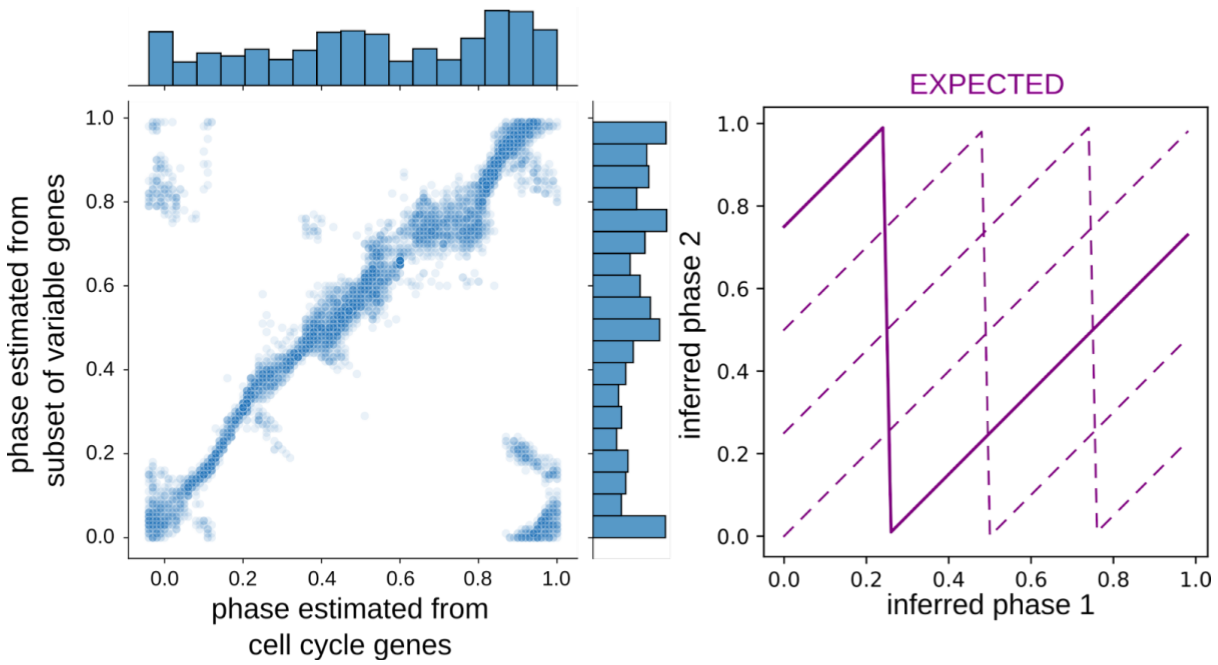

Supplementary Figure S6. DeepCycle is robust to change in the set of training genes. Transcriptional phases are estimated from the selected cell cycle genes (x-axis) and the set of highly variable genes (y-axis) in the mESC dataset. The two phases are highly correlated, showing the robustness of the results even with a lower number of genes. Since there is an arbitrary shift in the phases any of the patterns in the right panel correspond to a high correlation between the inferred phases. The histograms show that the distributions of inferred phases are uniform and there is no accumulation of cells at specific phases.

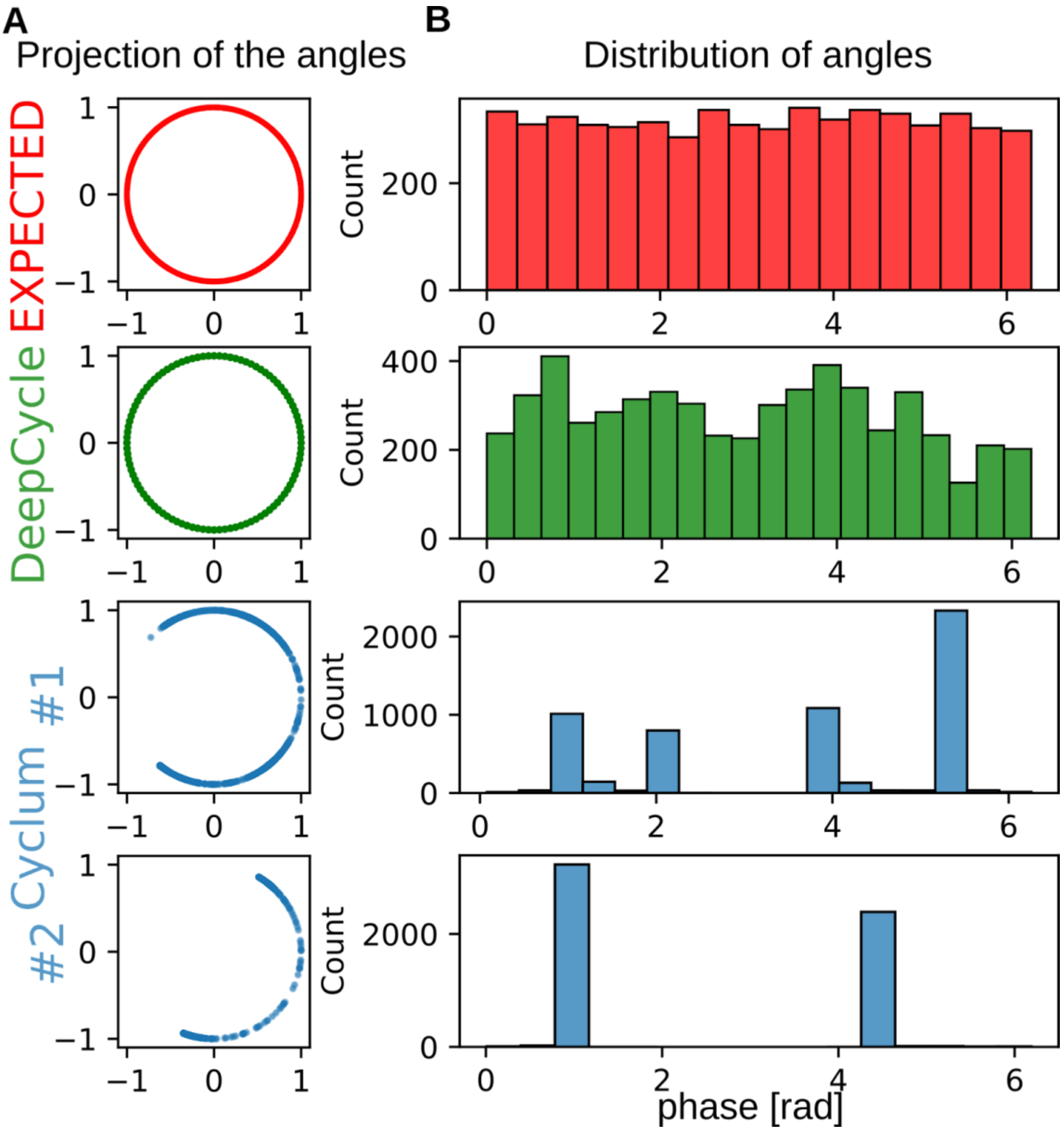

Supplementary Figure S7. Cyclum[7] is unstable and unable to find the correct cell cycle phase. Cyclum #1 and #2 represent two different runs of Cyclum with the same input. **A.** The angles inferred by Cyclum are not distributed uniformly around the circle but localize on the half circle, compare Cyclum vs EXPECTED and DeepCycle). **B.** The distribution of angles is also biased toward two opposite angles (Cyclum vs EXPECTED and DeepCycle). In conclusion the algorithm tries to separate the cell as much as possible, pushing them on the opposite sides of the circle. The expected behaviour would be a whole circle as in the EXPECTED and DeepCycle panel A (red and green) and an uniform distribution of angles in the top panel B, see the EXPECTED and DeepCycle rows. This analysis was performed on the mESC dataset.

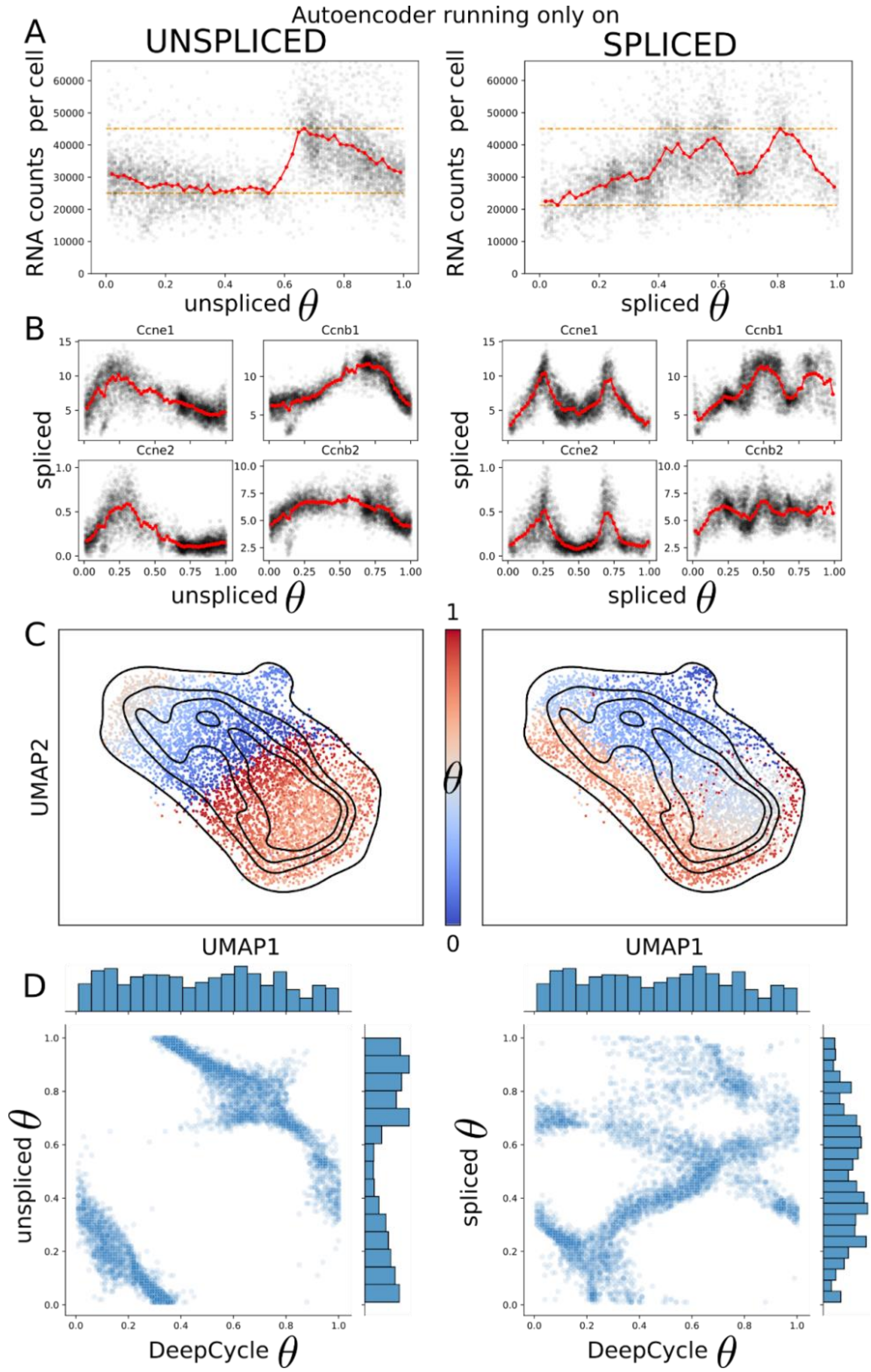

Supplementary Figure S8. Ablation analysis shows that unspliced and spliced reads alone give incorrect directionality and ordering of the cells. For the ablation analysis the part of the Autoencoder in the orange box in Supplementary Figure S3 has been removed. **A.** The RNA counts per cell are in the wrong directionality for the unspliced case and randomly going up and down for the spliced. **B.** Cyclins show the correct behaviour for the unspliced but the transcriptional phase is moving backwards. While for the unspliced there are multiple peaks in their expression. **C.** UMAP projections of the mESCs show the wrong directionality

given by the RNA velocity patterns and a totally inconsistent behaviour for the spliced case. **D.** Using the spliced as input the results become inconsistent with DeepCycle. While the unspliced capture the right phase but in the opposite direction.

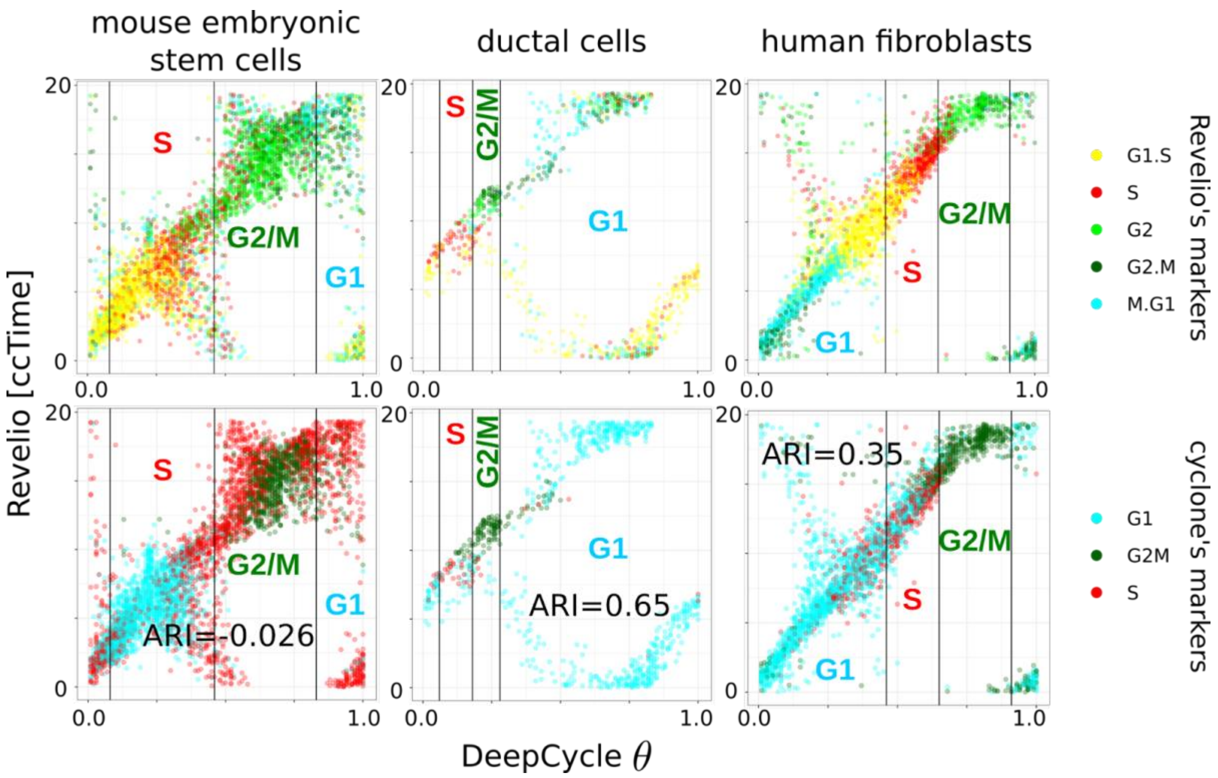

Supplementary Figure S9. Comparison of DeepCycle with Revelio[8] and Cyclone[29]. ccTime (cell cycle Time) from Revelio is used to compare with DeepCycle transcriptional phase (theta). The correspondence between DeepCycle's and Revelio's cell cycle progressions is quite striking and the markers used by Revelio for each subphase correspond to the phases defined by DeepCycle subsequent analysis. On the other hand, Cyclone is not able to correctly assign the cells to the phases in the mESCs (Adjusted Rand Index ARI=-0.026), while it looks more in line with DeepCycle and Revelio for the ductal cells (ARI=0.65) and the human fibroblasts (ARI=0.35).

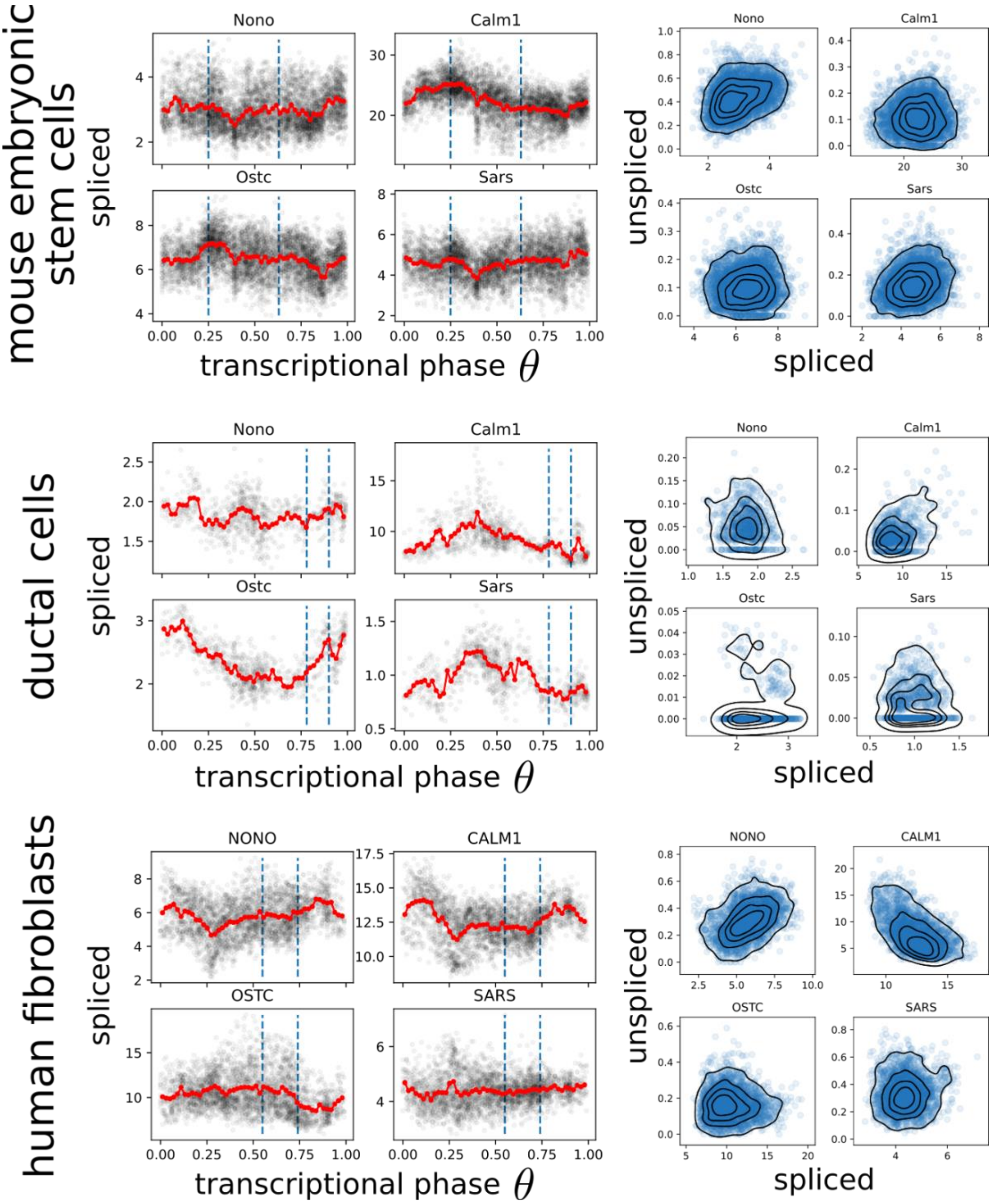

Supplementary Figure S10. Non-cycling genes. Examples of non-cycling genes along the transcriptional phase (left panel) and their respective unspliced-spliced patterns (right panel).

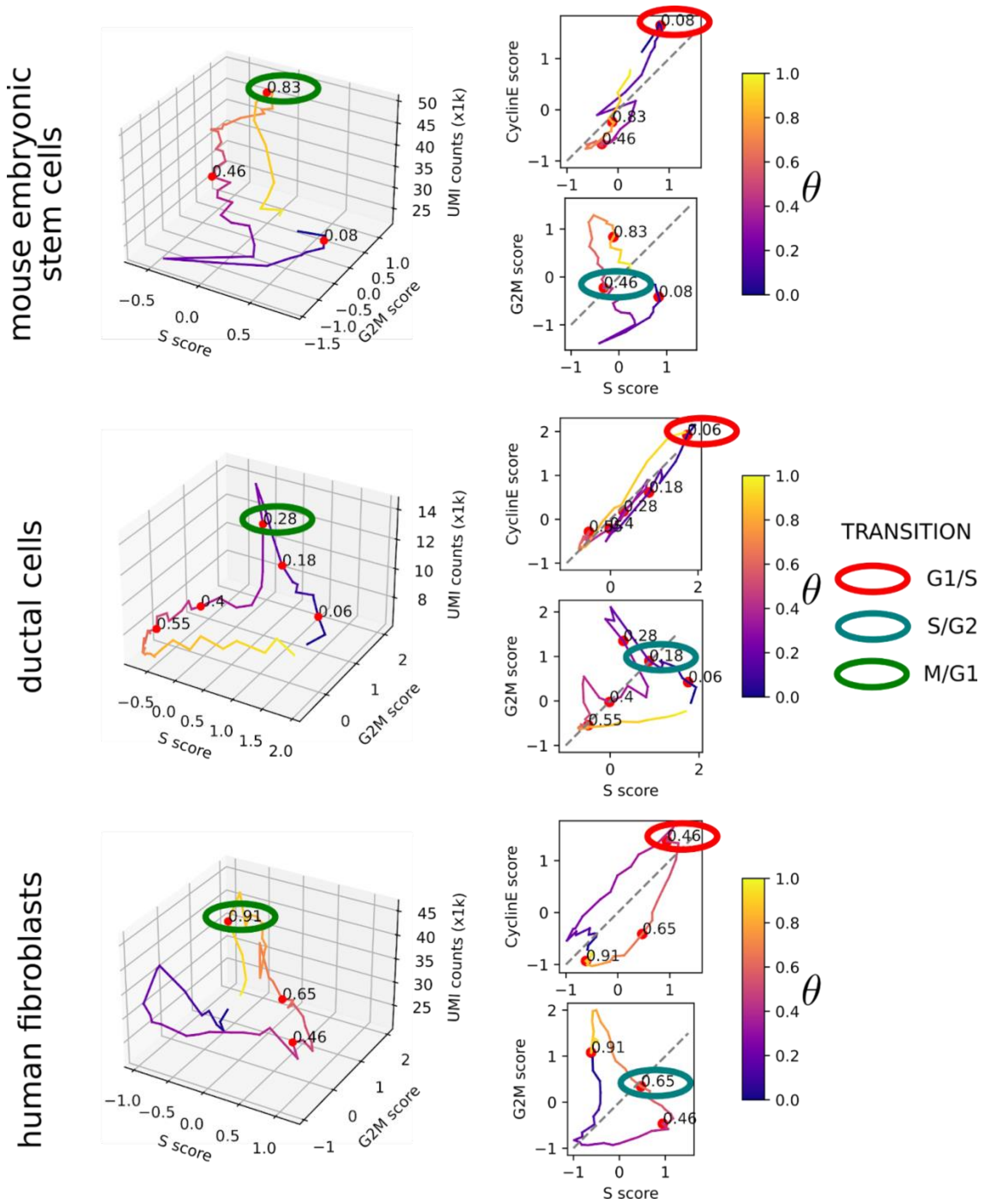

Supplementary Figure S11. Automated detection of the cell phase transitions. The transitions between the different cell cycle phases have been defined as follows. **M/G1** (green ellipses) are set at the first theta bin where the average number of counts drops (z-axis, UMI counts in the left panels). **G1/S** (red ellipses) are set at the peak in the cyclin E1 and E2 expressions, see Cyclin-E score in the Methods. The cyclin-E score reaches a peak slightly earlier than the S score, as expected since the cyclin-Es identify only the beginning of the S phase. **S/G2** (blue ellipses) are set at the theta where the G2M score becomes bigger than the S score. For the ductal cell, since the data are noisier, there are multiple potential S/G2, so we chose the only one in between the thetas for G1/S and M/G1 transitions. In this figure $\boldsymbol{\theta}$ is the raw transcriptional phase inferred from DeepCycle, for all the other figures the transcriptional phase have been translated to align the M/G1 transition to $\boldsymbol{\theta}$=1.

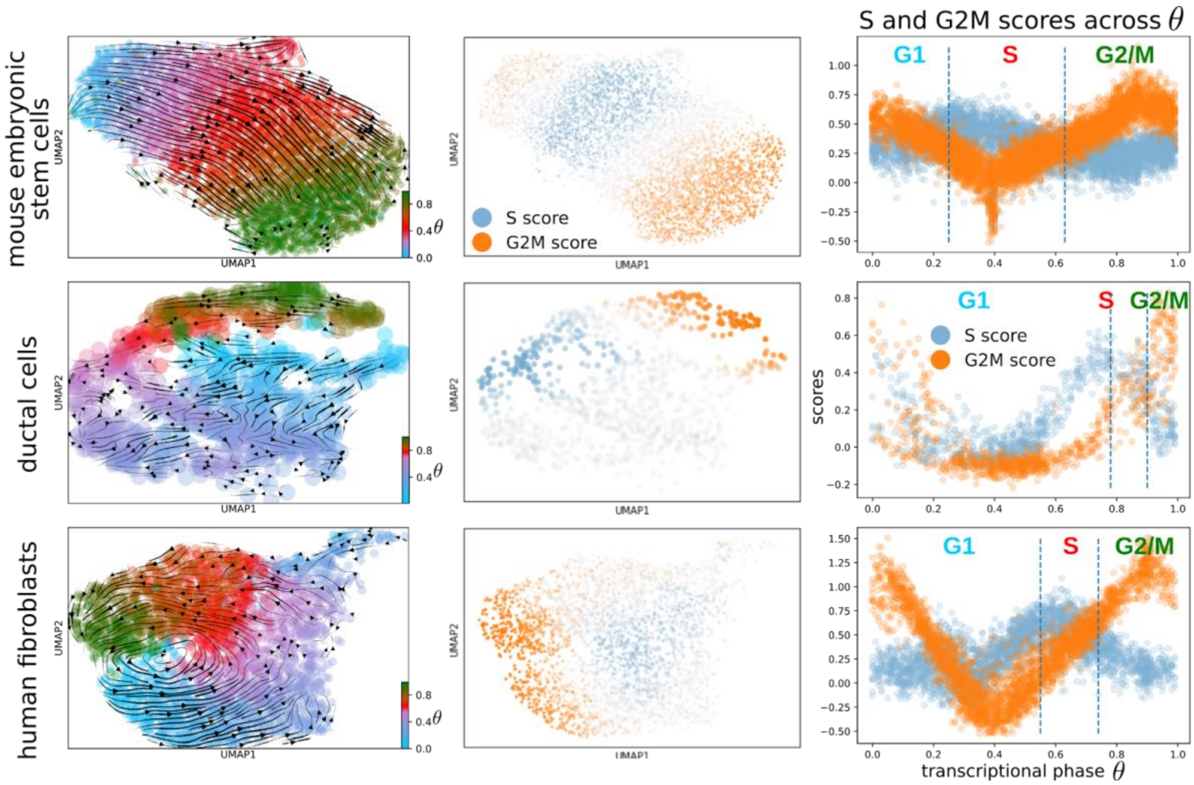

Supplementary Figure S12. (left panel) RNA velocity analysis for the mESCs, ductal cells, and the proliferative human fibroblasts. The genome-wide RNA velocity arrows follow the same direction identified by DeepCycle. **(center panel)** UMAP projections for mESCs, ductal cells, and human fibroblasts where cells are colored by their S and G2/M scores[19]. **(right panel)** The phases assigned through the analysis of DeepCycle results are consistent with the score cell cycle scores assigned by scVelo.

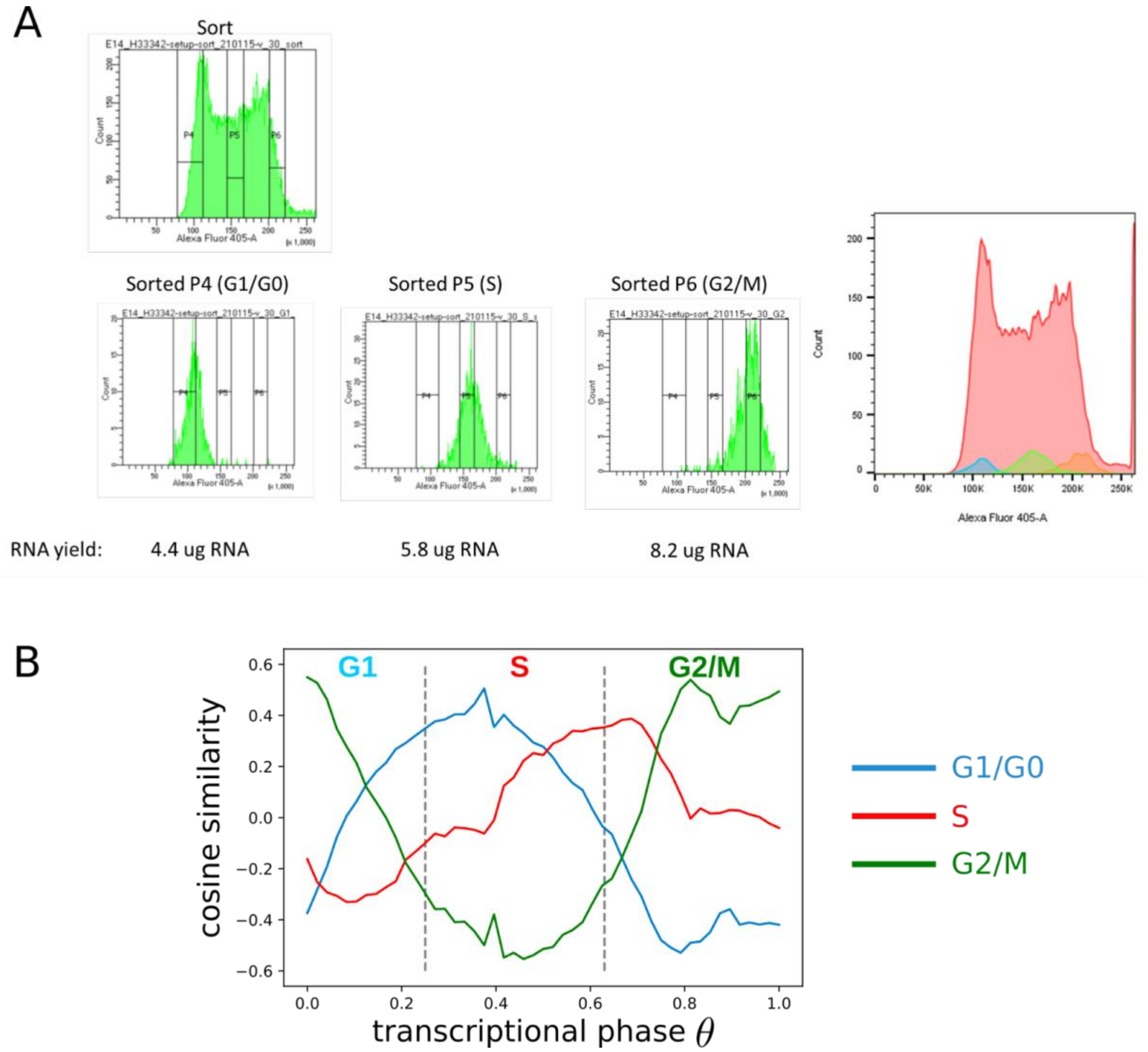

Supplementary Figure S13. Phase validation with FACS-sorted mESC bulk RNA-seqs. A. FAC sorting of E14Tg2a cells in different phases of the cell cycle. Top panel shows the cell cycle distribution of the whole population of Vybrant DyeCycle Violet-stained live singlets; P4, P5 and P6 depict the sorting gates for G1/G0, S and G2/M, respectively. Bottom left, middle and right panels show the sorted G1/G0, S and G2/M cells respectively, with respect to the original sorting gates. **B.** The cosine similarity has been calculated between the expression levels in the three facs-sorted populations (G1 in blue, S in red and G2M in green) and the average expression for each transcriptional phase in the mESCs. The three waves are consistent with our phase definition, though there is a shift of the G1/S transition perhaps due to a different definition of start of the S phase. Indeed for FACS sorted cells the S phase begins when DNA duplication has already started, for DeepCycle is set exactly at the cyclin peak, so presumably at an earlier cell cycle stage. This further elucidates the high resolution DeepCycle can reach.

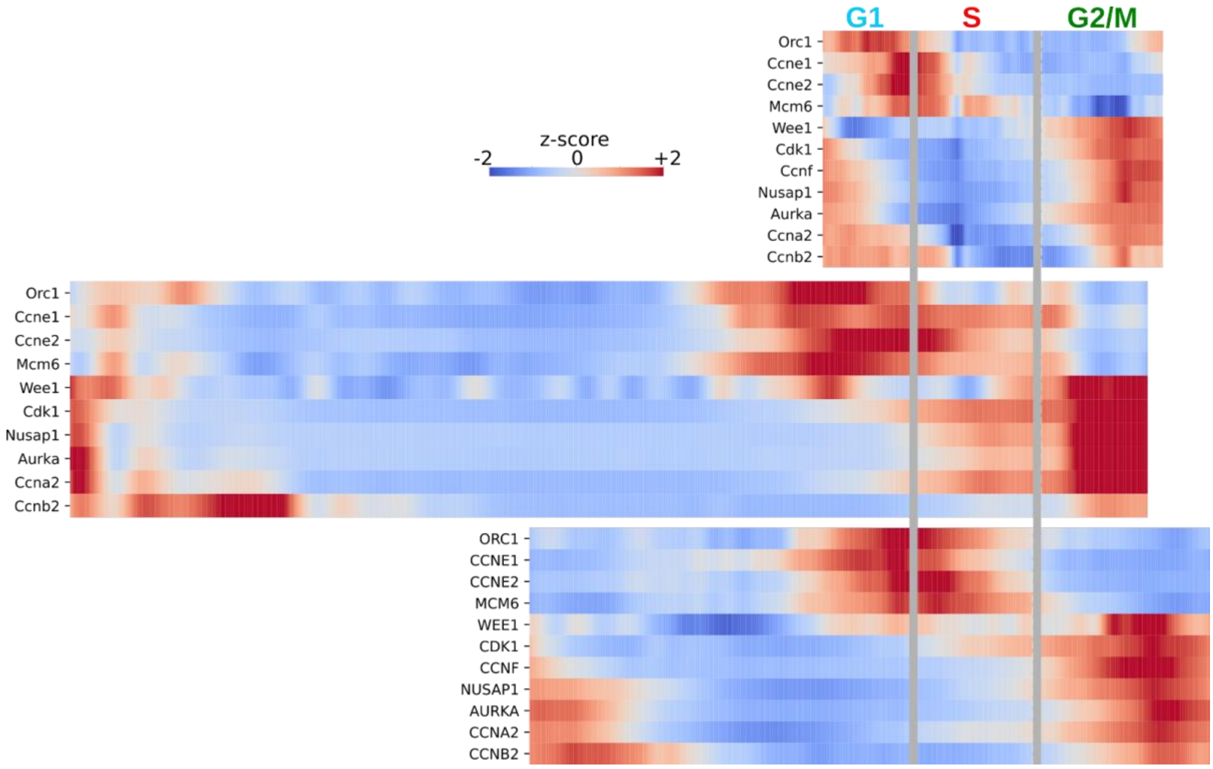

Supplementary Figure S14. Cell cycles on scale. By making the assumption that theta scales linearly with the time we can stretch the transcriptional phases, accordingly, to have the same length for the S phase in the three datasets. This transformation of theta shows the different lengths of the G1 phases across datasets with more differentiated cells with longer G1 phases and longer cell cycles. On the other side, the G2 and M phases are more consistent in length.

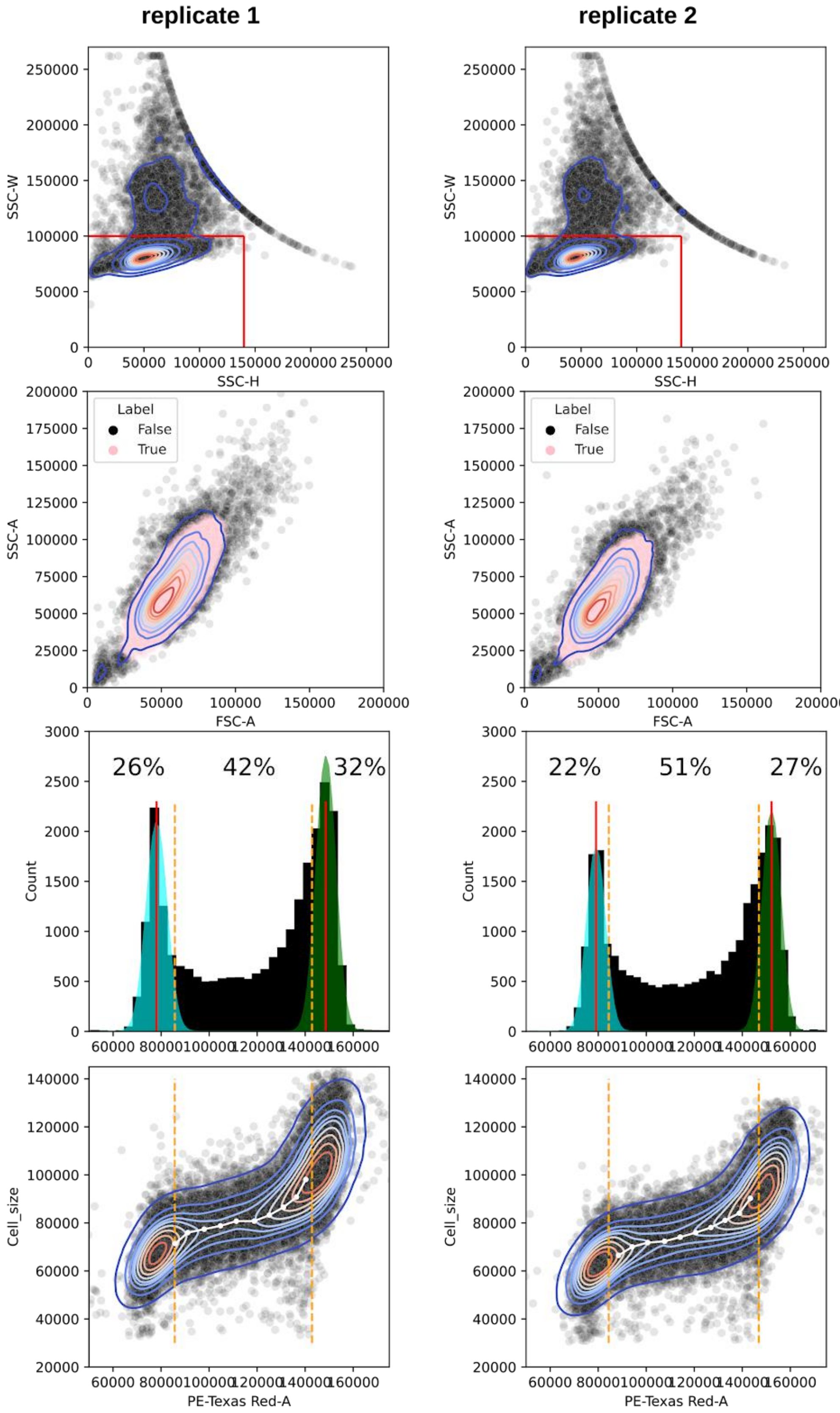

Supplementary Figure S15. Flow cytometry analysis. The true label shows the cells with the Hotelling T^2 lower than 6. Orange dashed lines delimit the G1/S and S/G2 transitions.

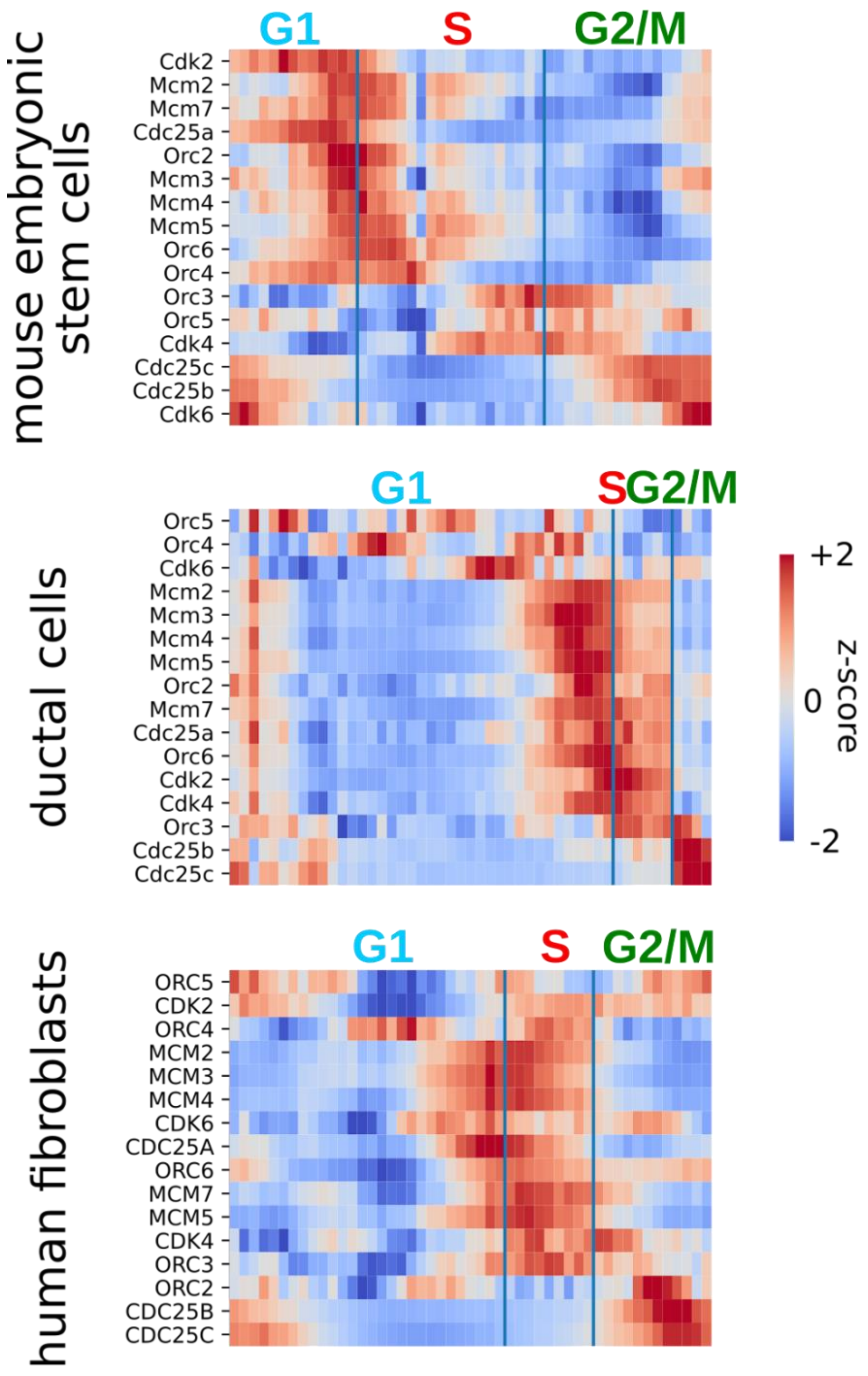

Supplementary Figure S16. Comparison of core cell cycle genes across datasets.

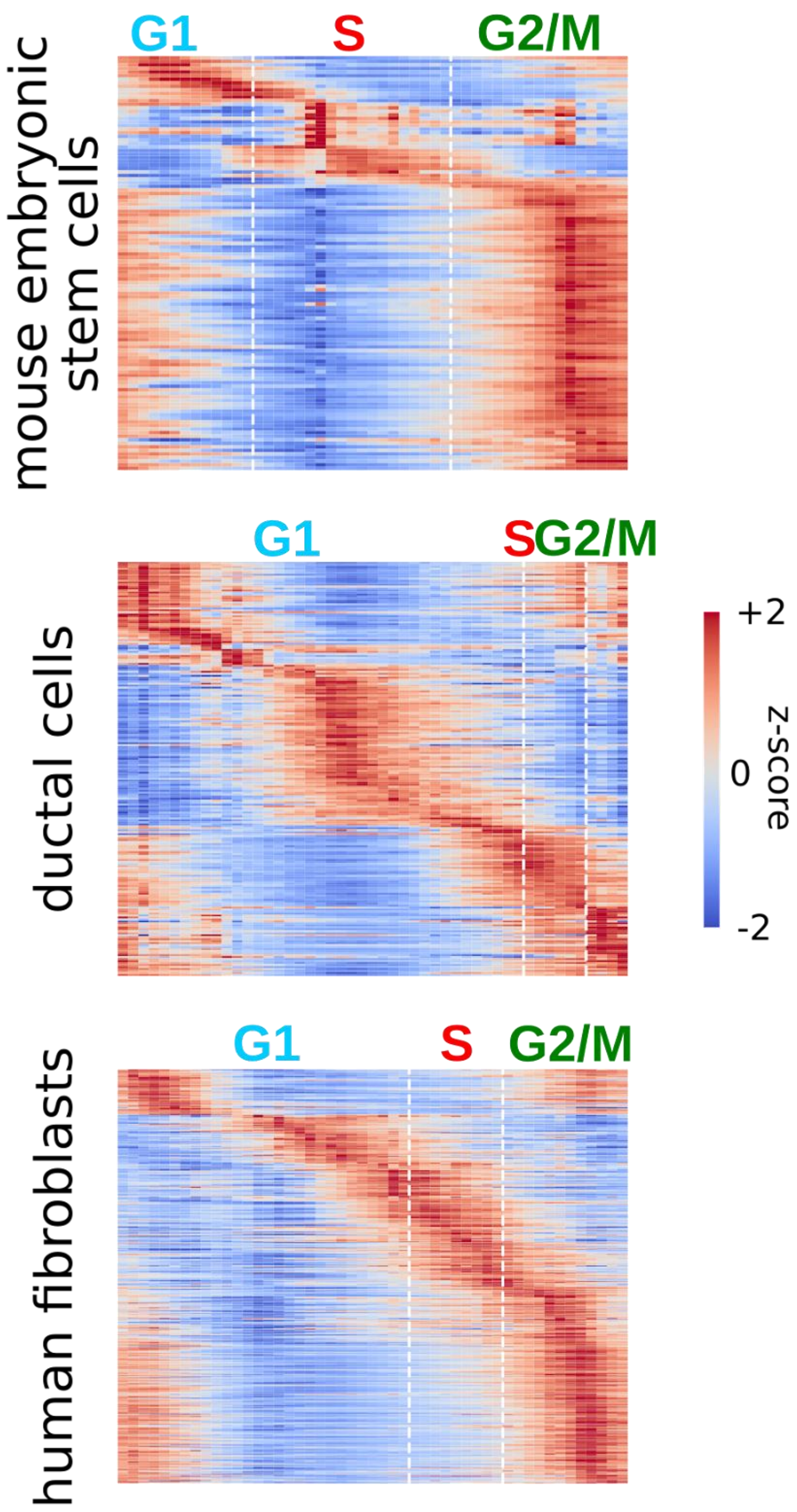

Supplementary Figure S17. Top genes selected based on their variability in expression across the cell cycle. By selecting only genes whose spliced expression is above 1, the majority of the genes in the 3 datasets are actually quite stable in expression levels across the cell cycle. Among these the genes showing 2-fold change across the cell cycle are 218 out of 790 for the ductal cells, 442 out of 3533 for the fibroblasts and 116 out of 4101 for the mESC.

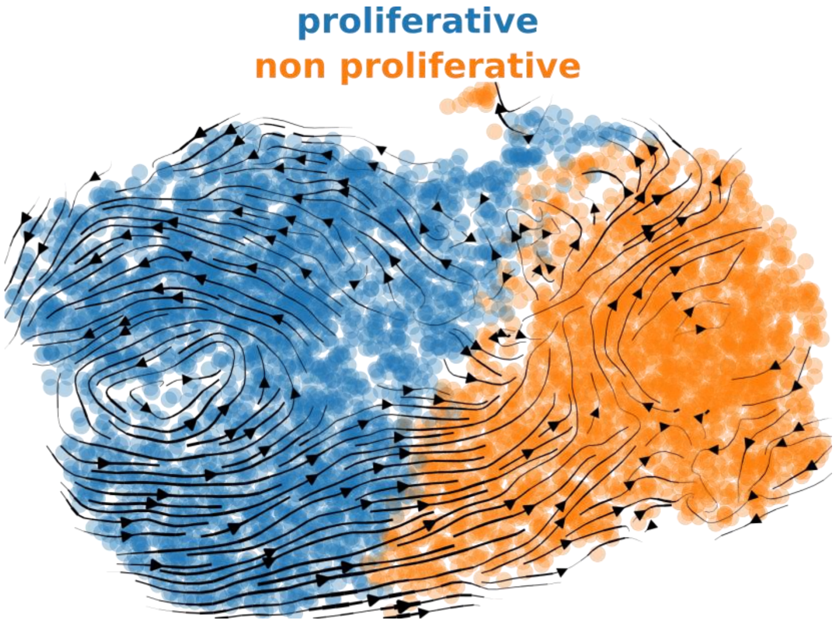

Supplementary Figure S18. RNA velocity analysis on the whole population of human fibroblasts. The cycling population shows a circular pattern of velocities, while the nonproliferative one looks more stable.

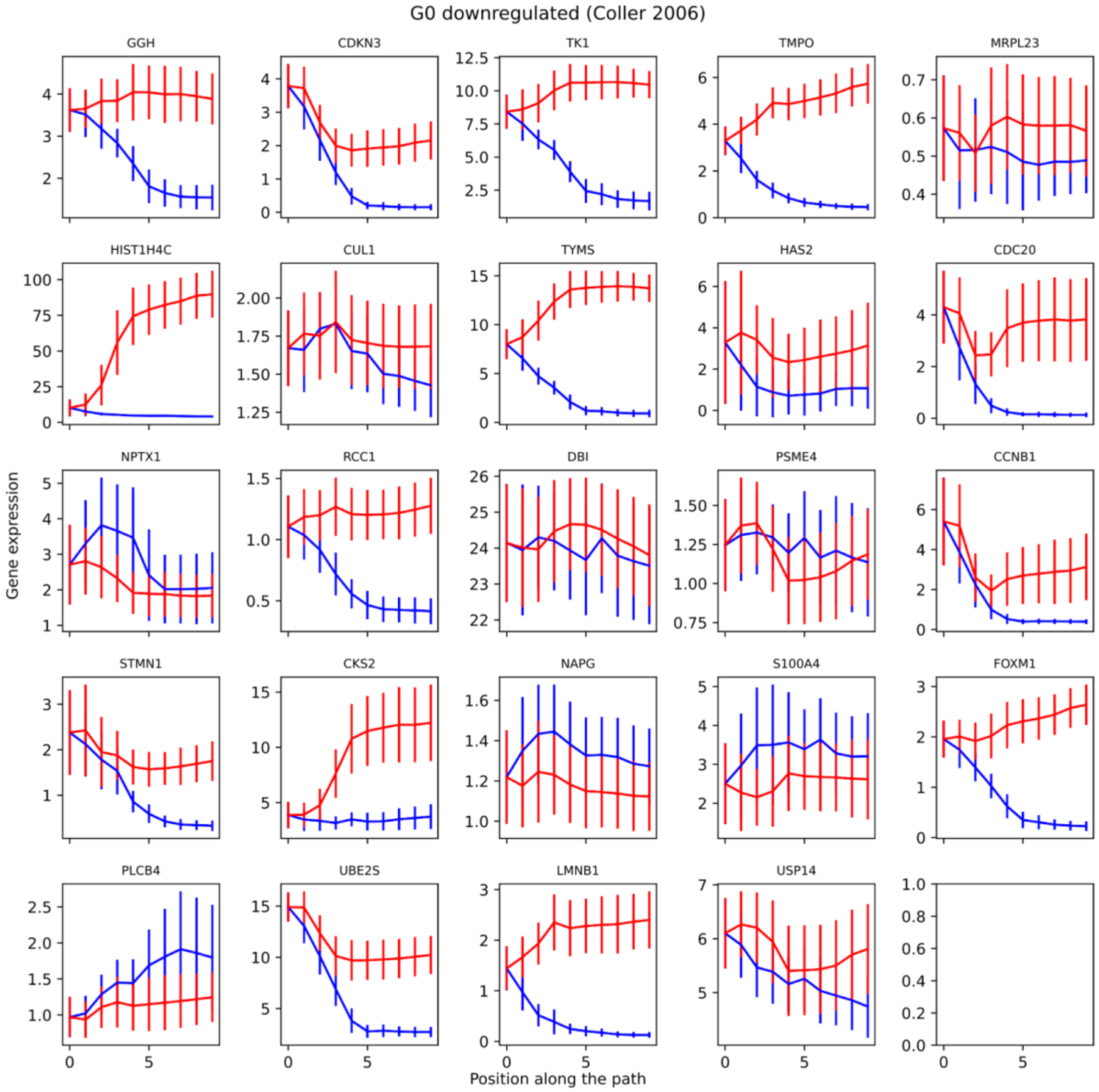

Supplementary Figure S19. Genes downregulated in G0 from Coller et al.[63] **are consistently downregulated in the nonproliferative fibroblasts**. The y-axis represents the average gene expression along the cell-cylce exit trayectory (blue) and G1-S transition (red). The x-axis represents the steps in the paths with the corresponding colors in Figure 5E. Averages were calculated pulling together between 100 to 500 cells in each step of the path (see Figure 5E). Vertical bars represent SDs.

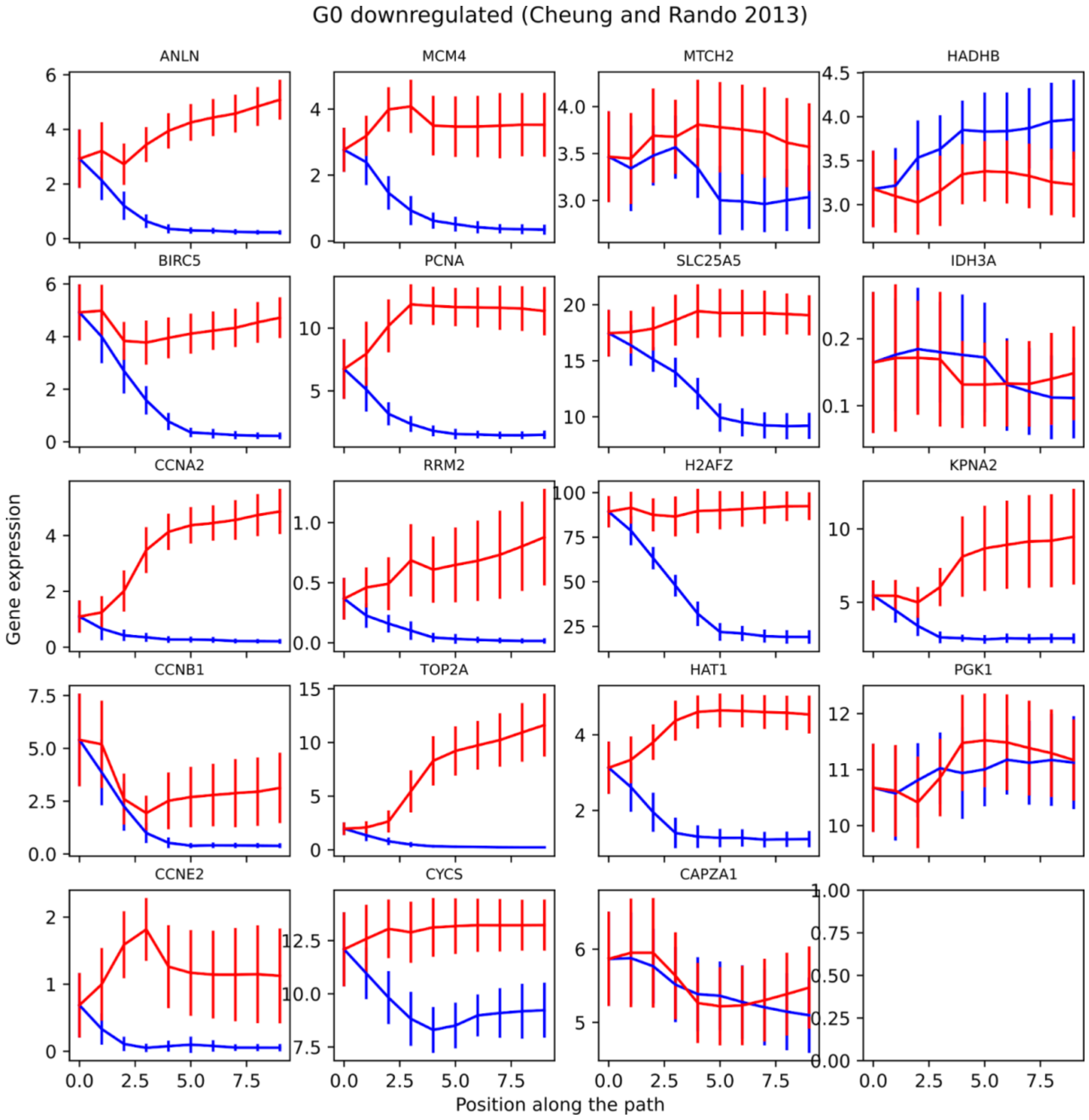

Supplementary Figure S20. Genes downregulated in G0 from Cheung and Rando [64] are consistently downregulated in the nonproliferative fibroblasts. The y-axis represents the average gene expression along the cell-cylce exit trayectory (blue) and G1-S transition (red). The x-axis represents the steps in the paths with the corresponding colors in Figure 5E. Averages were calculated pulling together between 100 to 500 cells in each step of the path (see Figure 5E). Vertical bars represent SDs.

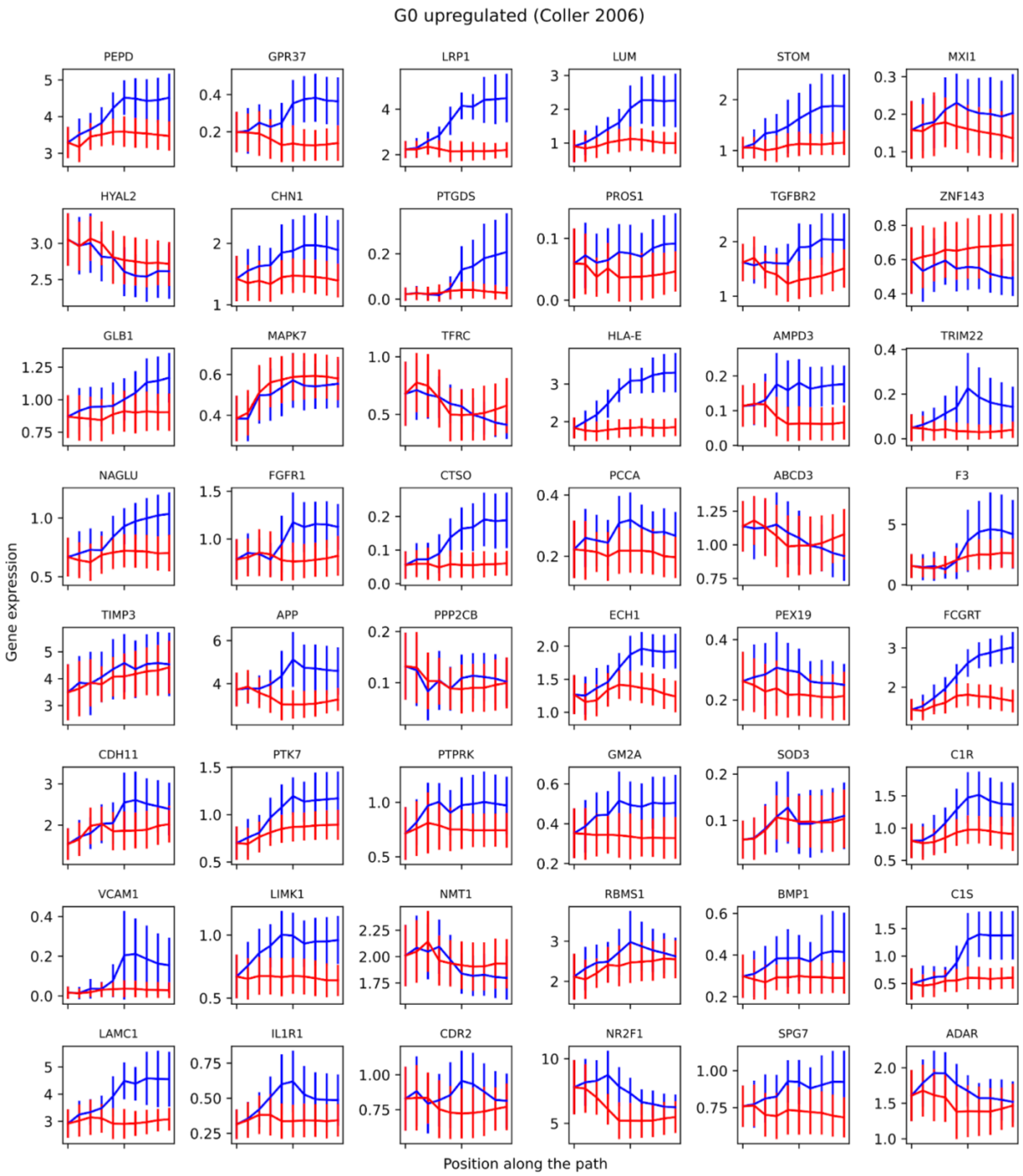

Supplementary Figure S21 (continue next page). Genes upregulated in G0 from Coller et al.[63] **are consistently upregulated in the nonproliferative fibroblasts**. The y-axis represents the average gene expression along the cell-cylce exit trayectory (blue) and G1-S transition (red). The x-axis represents the steps in the paths with the corresponding colors in Figure 5E. Averages were calculated pulling together between 100 to 500 cells in each step of the path (see Figure 5E). Vertical bars represent SDs.

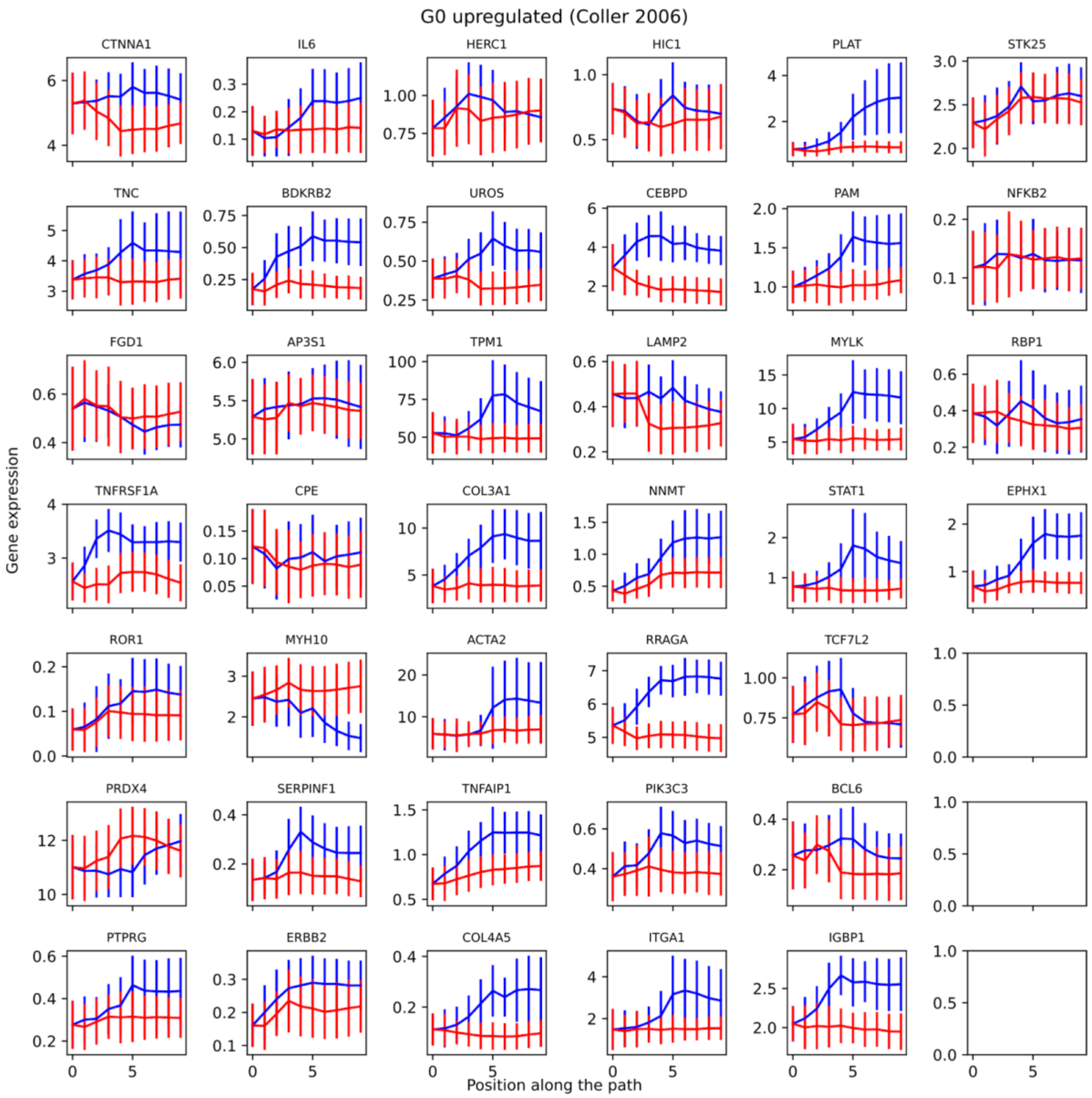

Supplementary Figure S21(bis). Genes upregulated in G0 from Coller et al.[63] are consistently upregulated in the nonproliferative fibroblasts. The y-axis represents the average gene expression along the cell-cylce exit trajectory (blue) and G1-S transition (red). The x-axis represents the steps in the paths with the corresponding colors in Figure 5E. Averages were calculated pulling together between 100 to 500 cells in each step of the path (see Figure 5E). Vertical bars represent SDs.

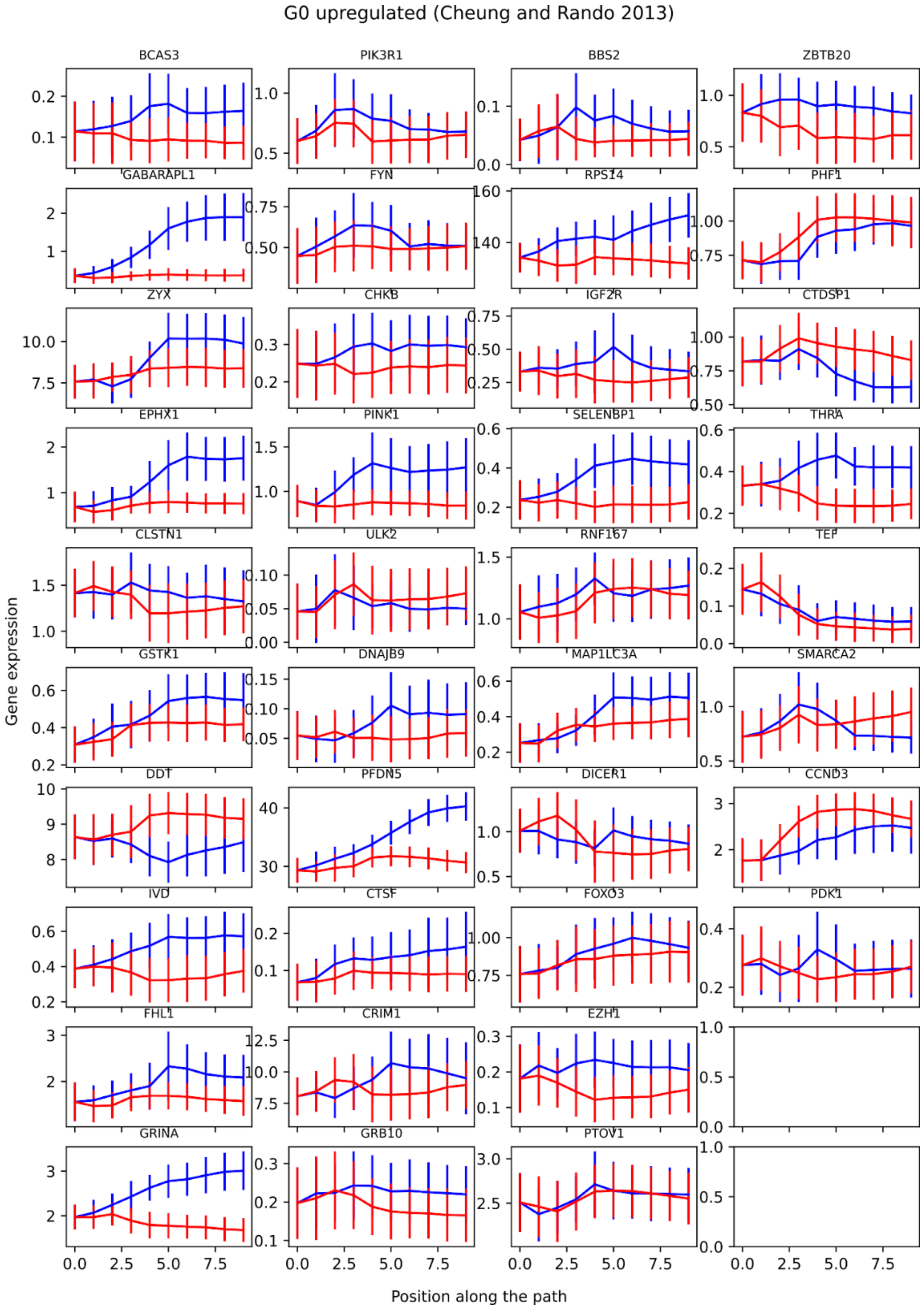

Supplementary Figure S22. Genes upregulated in G0 from Cheung and Rando[64] are consistently upregulated in the nonproliferative fibroblasts. The y-axis represents the average gene expression along the cell-cylce exit trayectory (blue) and G1-S transition (red). The x-axis represents the steps in the paths with the corresponding colors in Figure 5E. Averages were calculated pulling together between 100 to 500 cells in each step of the path (see Figure 5E). Vertical bars represent SDs.

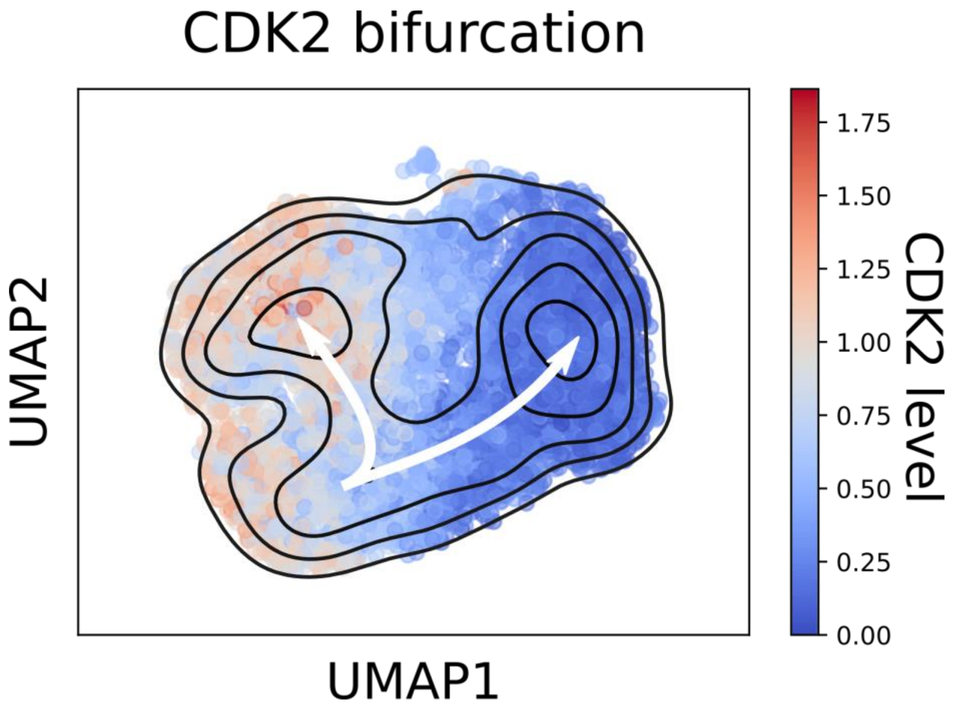

Supplementary Figure S23. CDK2 bifurcation. CDK2 has been observed as crucial for the proliferation-quiescence decision[67]. Consistently, CDK2 levels decrease and stay low for cells transitioning to a quiescent state, and vice versa it increases for cells proceeding towards S phase and a new cycle.

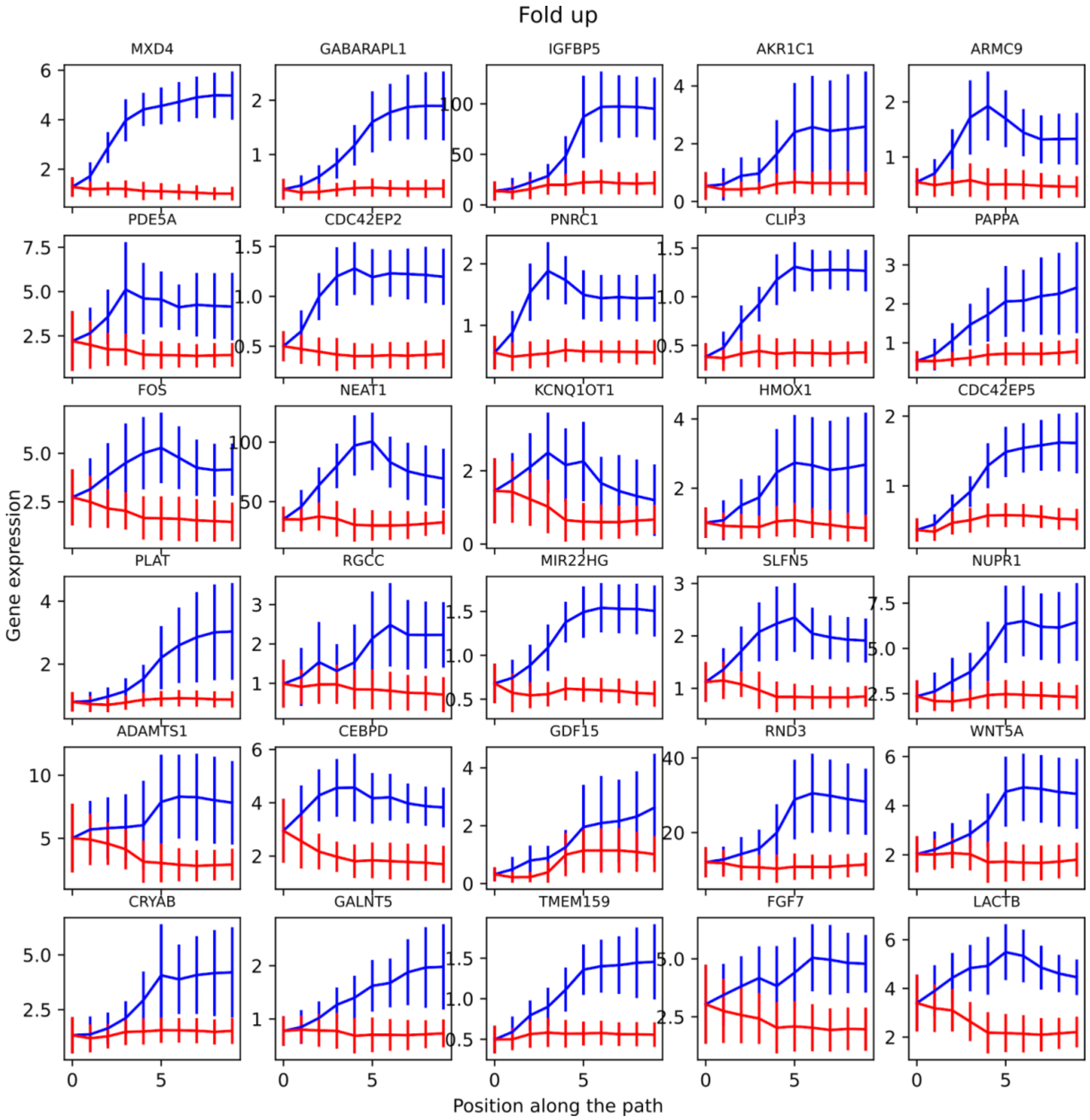

Supplementary Figure S24. Most upregulated genes during the transition from G1 to quiescence-like state. Top genes showing the greatest cumulative fold-up change towards the nonproliferative state. The y-axis represents the average gene expression along the cell-cylce exit trayectory (blue) and G1-S transition (red). The x-axis represents the steps in the paths with the corresponding colors in Figure 5E. Averages were calculated pulling together between 100 to 500 cells in each step of the path (see Figure 5E). Vertical bars represent SDs.

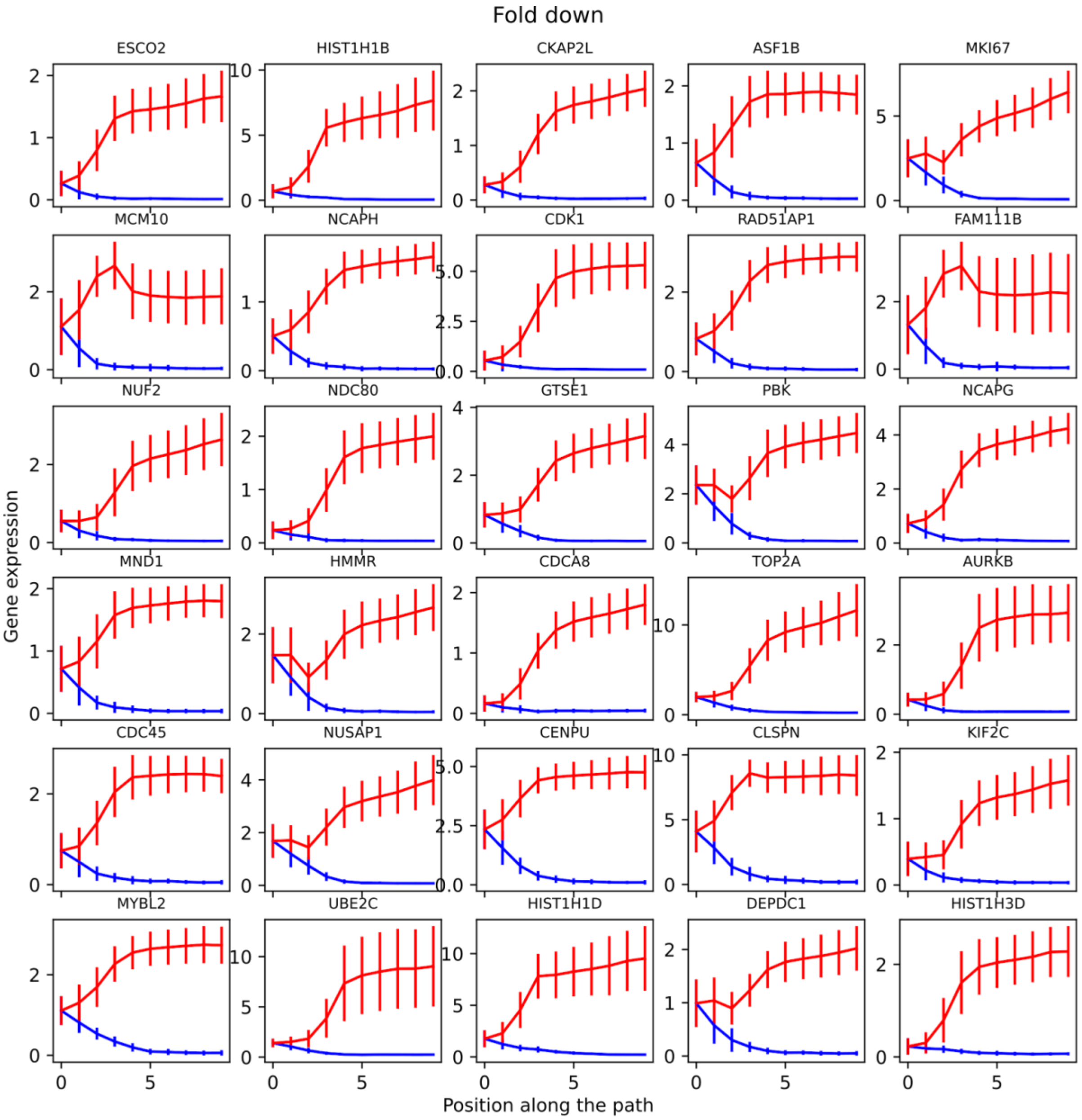

Supplementary Figure S25. Most upregulated genes during the transition from G1 to S phase. Top genes showing the greatest cumulative fold-down change towards the nonproliferative state. The y-axis represents the average gene expression along the cell-cylce exit trayectory (blue) and G1-S transition (red). The x-axis represents the steps in the paths with the corresponding colors in Figure 5E. Averages were calculated pulling together between 100 to 500 cells in each step of the path (see Figure 5E). Vertical bars represent SDs.

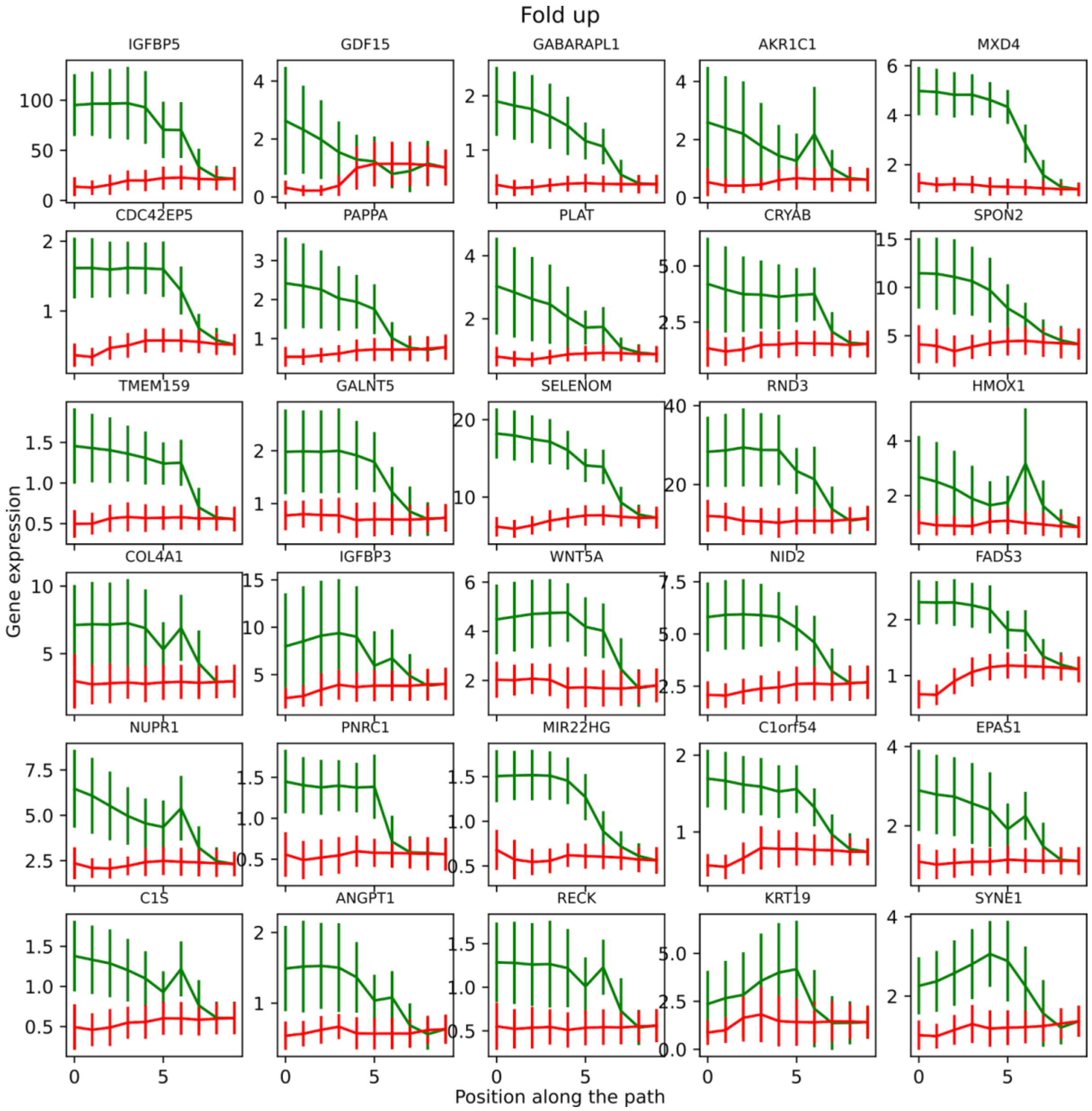

Supplementary Figure S26. Most upregulated genes during the hypothetical transition from the quiescence-like state to S phase. Top genes showing the greatest cumulative fold-up change towards the S phase. The y-axis represents the average gene expression along the cell-cylce reentry trayectory (top curves in green) and G1-S transition (bottom curves in red). The x-axis represents the steps in the paths with the corresponding colors in Figure 5E. Averages were calculated pulling together between 100 to 500 cells in each step of the path (see Figure 5E). Vertical bars represent SDs.

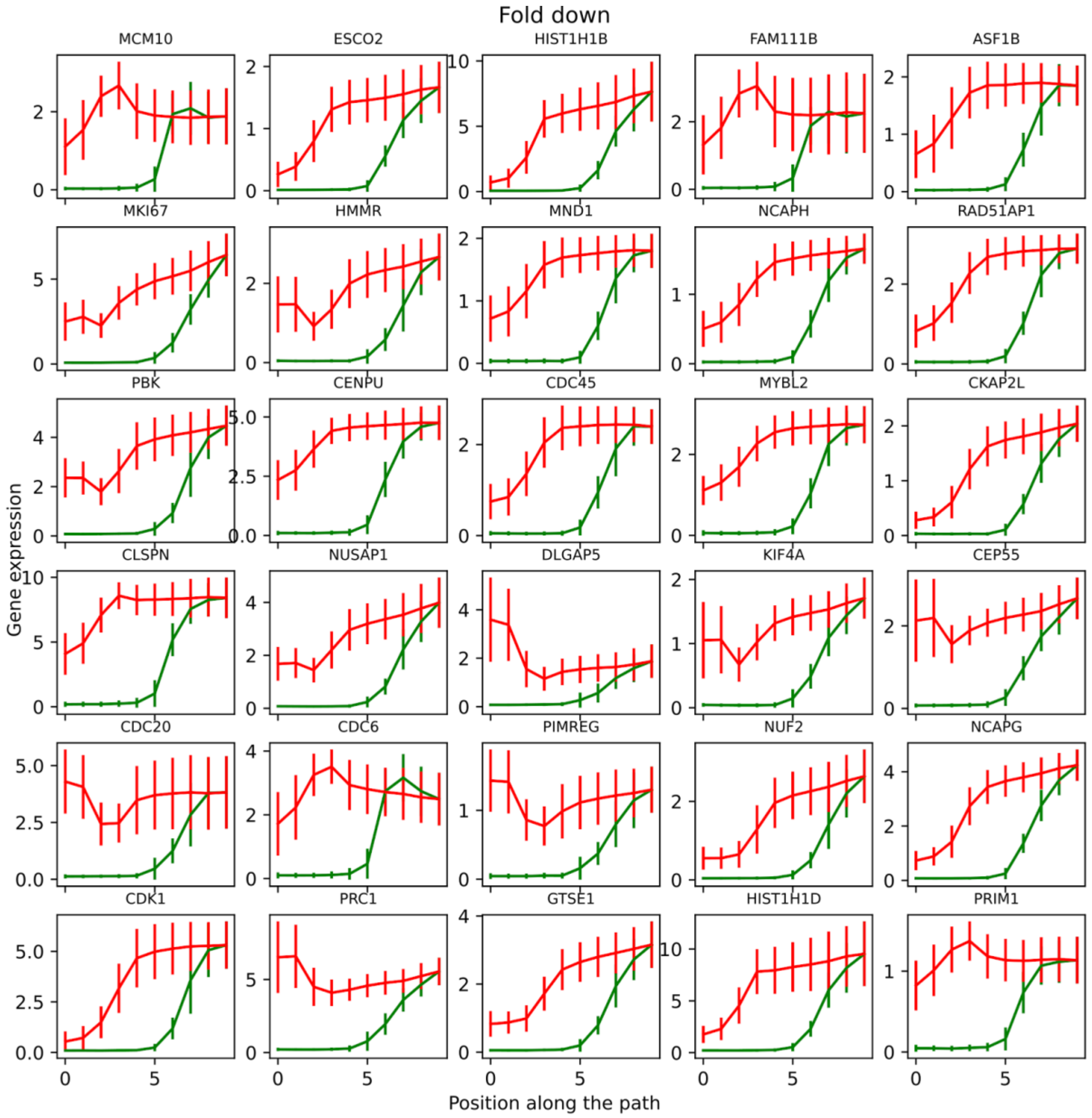

Supplementary Figure S27. Most downregulated genes during the hypothetical transition from the quiescence-like state (green) to S phase. Top genes showing the greatest cumulative fold-down change towards the S phase. The y-axis represents the average gene expression along the G1-S transition (top curves in red) and cell-cylce reentry trayectory (bottom curves in green). The x-axis represents the steps in the paths with the corresponding colors in Figure 5E. Averages were calculated pulling together between 100 to 500 cells in each step of the path (see Figure 5E). Vertical bars represent SDs.

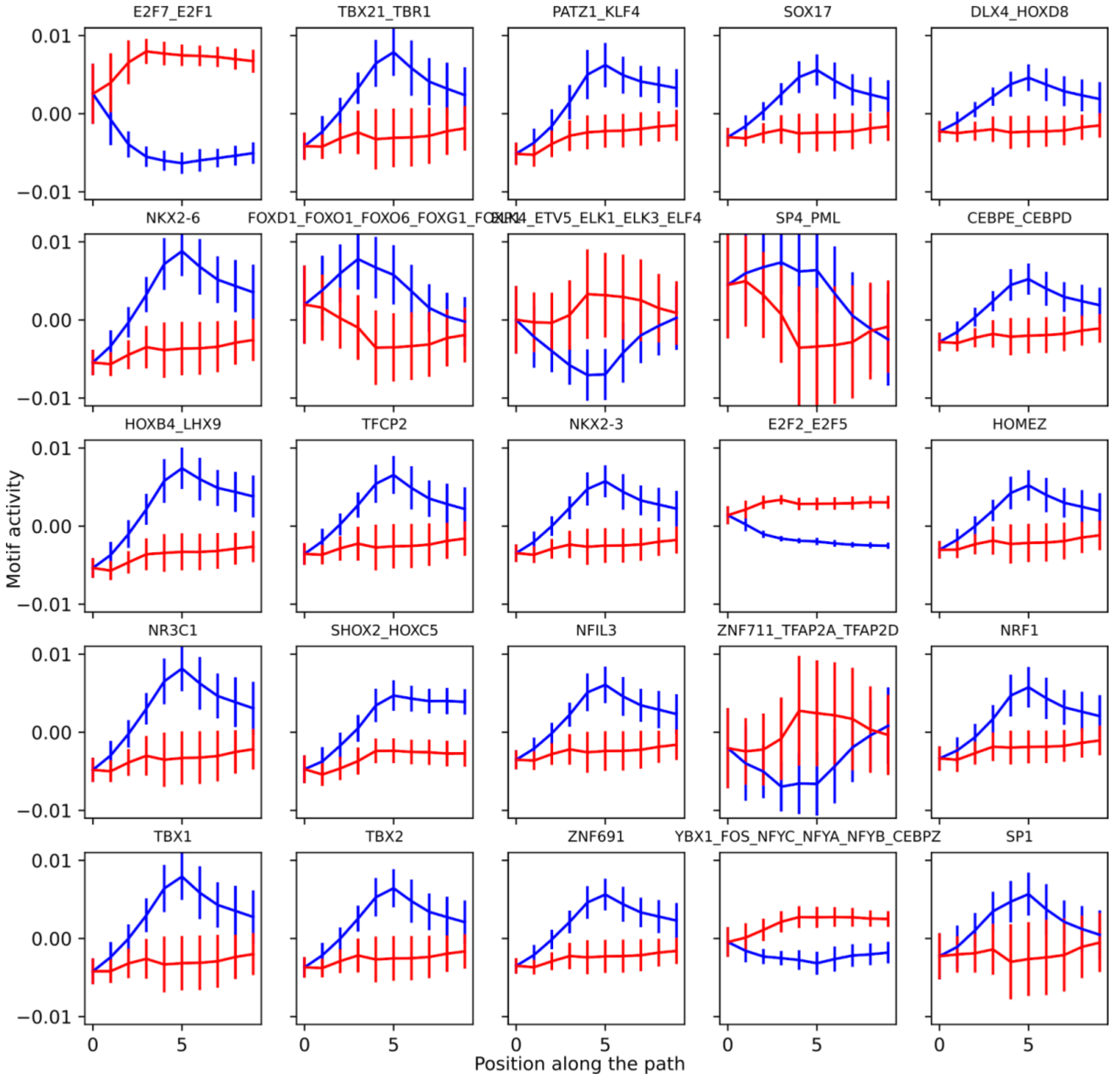

Supplementary Figure S28. Most significant motifs distinguishing the paths leaving from G1. The y-axis represents the average motif activity along the cell-cylce exit trayectory (blue) and G1-S transition (red). The x-axis represents the steps in the paths with the corresponding colors in Figure 5E. Averages were calculated pulling together between 100 to 500 cells in each step of the path (see Figure 5E). Vertical bars represent SDs.

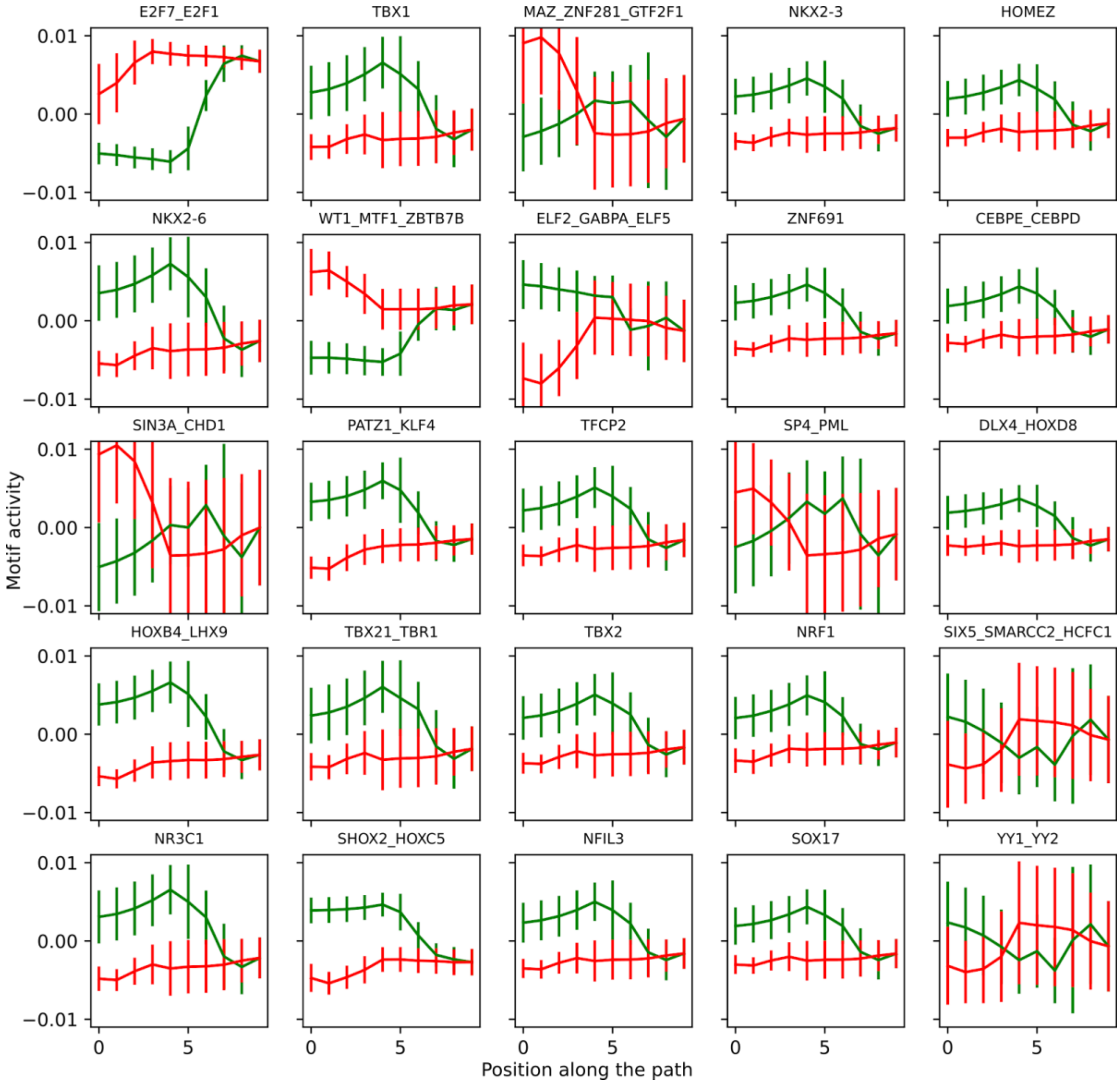

Supplementary Figure S29. Most significant motifs distinguishing the paths towards S. The y-axis represents the average motif activity along the cell-cylce reentry trayectory (in green) and G1-S transition (in red). The x-axis represents the steps in the paths with the corresponding colors in Figure 5E. Averages were caluclated pulling together between 100 to 500 cells in each step of the path (see Figure 5E) and vartical bars represent SDs.
