# Supplementary material for: Cell cycle gene regulation dynamics revealed by RNA velocity and deep-learning
Source: Nat Commun. 2022 May 23;13:2865. doi: 10.1038/s41467-022-30545-8 (PMC9126911; doi:10.1038/s41467-022-30545-8)
Supplement: Supplementary file 2 — Reporting Summary [file 41467_2022_30545_MOESM2_ESM.pdf]

nature research

Corresponding author(s): Andrea Riba, Nacho Molina

Last updated by author(s): Apr 25, 2022

Reporting Summary

Nature Research wishes to improve the reproducibility of the work that we publish. This form provides structure for consistency and transparency in reporting. For further information on Nature Research policies, see our Editorial Policies and the Editorial Policy Checklist.

Statistics

For all statistical analyses, confirm that the following items are present in the figure legend, table legend, main text, or Methods section.

n/a	Confirmed	
☐	☒	The exact sample size (n) for each experimental group/condition, given as a discrete number and unit of measurement
☐	☒	A statement on whether measurements were taken from distinct samples or whether the same sample was measured repeatedly
☐	☒	The statistical test(s) used AND whether they are one- or two-sided<br>*Only common tests should be described solely by name; describe more complex techniques in the Methods section.*
☒	☐	A description of all covariates tested
☒	☐	A description of any assumptions or corrections, such as tests of normality and adjustment for multiple comparisons
☒	☐	A full description of the statistical parameters including central tendency (e.g. means) or other basic estimates (e.g. regression coefficient) AND variation (e.g. standard deviation) or associated estimates of uncertainty (e.g. confidence intervals)
☐	☒	For null hypothesis testing, the test statistic (e.g. F, t, r) with confidence intervals, effect sizes, degrees of freedom and P value noted<br>*Give P values as exact values whenever suitable.*
☒	☐	For Bayesian analysis, information on the choice of priors and Markov chain Monte Carlo settings
☒	☐	For hierarchical and complex designs, identification of the appropriate level for tests and full reporting of outcomes
☐	☒	Estimates of effect sizes (e.g. Cohen's d, Pearson's r), indicating how they were calculated

Our web collection on statistics for biologists contains articles on many of the points above.

Software and code

Policy information about availability of computer code

Data collection	DeepCycle is stored at https://github.com/andreariba/DeepCycle
Data analysis	the following packages have been used to perform the analysis:<br>python 3.7.9<br>scipy 1.5.2<br>numpy 1.19.1<br>pandas 1.1.1<br>scikit-learn 0.23.2<br>tensorflow 2.2.0<br>anndata 0.7.4<br>matplotlib 3.3.1<br>seaborn 0.10.1<br>scanpy 1.4.4.post1<br>scvelo 0.2.2<br>velocyto 0.17.17

For manuscripts utilizing custom algorithms or software that are central to the research but not yet described in published literature, software must be made available to editors and reviewers. We strongly encourage code deposition in a community repository (e.g. GitHub). See the Nature Research guidelines for submitting code & software for further information.

Data

Policy information about availability of data

All manuscripts must include a data availability statement. This statement should provide the following information, where applicable:

- Accession codes, unique identifiers, or web links for publicly available datasets
- A list of figures that have associated raw data
- A description of any restrictions on data availability

The raw data generated in this study has been deposited in the GEO database under accession code GSE167609. The binding site predictions used in this study are available in the SwissRegulon database (https://swissregulon.unibas.ch/sr/downloads). The source data files with the results of this paper are openly accessible in Zenodo under the DOI 10.1101/2021.03.17.43588786.

Field-specific reporting

Please select the one below that is the best fit for your research. If you are not sure, read the appropriate sections before making your selection.

[x] Life sciences [ ] Behavioural & social sciences [ ] Ecological, evolutionary & environmental sciences

For a reference copy of the document with all sections, see nature.com/documents/nr-reporting-summary-flat.pdf

Life sciences study design

All studies must disclose on these points even when the disclosure is negative.

Sample size	No sample size was chosen in advance.
Data exclusions	Data were not excluded from the analysis.
Replication	All the computational findings of this study were reproducible. scRNA-seq experiments were not replicated for luck of funding.
Randomization	Randomization was not relevant in this study as controlling for confounding factors was not required.
Blinding	This study did not involve any clinical research thus blinding was not relevant and was not used.

Reporting for specific materials, systems and methods

We require information from authors about some types of materials, experimental systems and methods used in many studies. Here, indicate whether each material, system or method listed is relevant to your study. If you are not sure if a list item applies to your research, read the appropriate section before selecting a response.

Materials & experimental systems

n/a	Involved in the study	
[x]	[ ]	Antibodies
[ ]	[x]	Eukaryotic cell lines
[x]	[ ]	Palaeontology and archaeology
[x]	[ ]	Animals and other organisms
[x]	[ ]	Human research participants
[x]	[ ]	Clinical data
[x]	[ ]	Dual use research of concern

Methods

n/a	Involved in the study	
[x]	[ ]	ChIP-seq
[ ]	[x]	Flow cytometry
[x]	[ ]	MRI-based neuroimaging

Eukaryotic cell lines

Policy information about cell lines

Cell line source(s)	The mouse ES E14Tg2a cell line is from the ECACC General Cell Collection; catalogue number: 08021401; The primary human fibroblasts IMR90 are from CORIELL Institute for Medical Research, Reference: I90-19
Authentication	Mouse ES cells were periodically verified for ES cell-like morphology. Human primary fibroblasts were used in low passage number and showed proper fibroblast morphology as well as proliferative capacity.
Mycoplasma contamination	Mouse ES cells were mycoplasm free. Human fibroblasts were regularly tested negative for the presence of mycoplasm.
Commonly misidentified lines (See ICLAC register)	No commonly misidentified cell lines were used in this study.

Flow Cytometry

Plots

Confirm that:

- [x] The axis labels state the marker and fluorochrome used (e.g. CD4-FITC).
- [x] The axis scales are clearly visible. Include numbers along axes only for bottom left plot of group (a 'group' is an analysis of identical markers).
- [x] All plots are contour plots with outliers or pseudocolor plots.
- [x] A numerical value for number of cells or percentage (with statistics) is provided.

Methodology

Sample preparation	Cells were harvested by trypsin as before and washed once with PBS. About 2 million cells were resuspended in 100 µl PBS and added drop-by-drop to 900 µl 95 % ethanol while mixing, then stored at +4°C overnight. Cells were then collected by centrifugation, washed once with PBS, re-suspended in 1 ml staining buffer (50 µg/ml propidium iodide, 2 mM MgCl2-, 50 ng/ml RNaseA [EN0531, ThermoScientific] in PBS) and incubated for 20 minutes at 37°C. Stained cells were washed once with PBS.
Instrument	BD LSRII flow cytometer
Software	The fcs files were processed with fcsparser (https://github.com/eyurtsev/fcsparser).
Cell population abundance	The filtering retained more than 80% of the original cells (~ 26k out of 31k).
Gating strategy	For the mESCs, the debris in the data was removed by filtering SSC-H and SSC-W values higher than 140000 and 100000, respectively, and by selecting cells with a Hotelling T2 value lower than 6 in the FSC-A SSC-A space, see Supplementary Figure S8. For the human fibroblasts, SSC-H values lower than 25000 and, SSC-H and SSC-W values greater than 150000 and 110000, respectively, were excluded. As for the mESCs, only cells with Hotelling T2 lower than 6 in the FSC-A SSC-A space were retained.

- [x] Tick this box to confirm that a figure exemplifying the gating strategy is provided in the Supplementary Information.
