# Supplementary material for: Cell cycle gene regulation dynamics revealed by RNA velocity and deep-learning
Source: Nat Commun. 2022 May 23;13:2865. doi: 10.1038/s41467-022-30545-8 (PMC9126911; doi:10.1038/s41467-022-30545-8)
Supplement: Supplementary file 3 — Description of Additional Supplementary Files [file 41467_2022_30545_MOESM3_ESM.pdf]

Title: Supplementary Data 1

Description: List of genes (n=158) presenting multiple maxima in the unspliced-spliced RNA space among which 13 are not yet considered in the GO term:cell_cycle and could be added as potential markers of the cell cycle.
